# Supplementary material for: Optical widefield nuclear magnetic resonance microscopy
Source: Nat Commun. 2025 Feb 3;16:1281. doi: 10.1038/s41467-024-55003-5 (PMC11790880; doi:10.1038/s41467-024-55003-5)
Supplement: Supplementary file 1 — Supplementary Information [file 41467_2024_55003_MOESM1_ESM.pdf]

Supplementary Information

Supplementary Note 1

Statistical vs. Boltzmann polarized NMR

NV based NMR techniques may be separated into two categories based on the nature of the liquid state NMR signal they detect.

1. **Statistical NMR signals:** A finite (open, sensing) volume, containing thermalized sample spins, will spontaneously emit short-lived NMR signals. These signals arise from random fluctuations in the average spin orientation and have a lifetime limited by the average diffusion time of the sample spins through the dominant detection volume. As the sample spins present in the detection volume exchange with the surrounding sample, the cumulative spin polarization decays and may randomly reappear with little or no correlation to the previous signal. The line widths of NMR spectra of this origin are thus limited to several kilohertz, depending on the sample diffusion coefficient and NV implantation depth (See Supplementary Figure 1). We approximate the expected linewidth according to Eq. 1 where τ is the average diffusion time through the detection volume as a function of the sample's diffusion coefficient (D) and the NV implantation depth.

$$\Gamma = \frac{1}{\pi \cdot \tau(D, \mathrm{NV} - \mathrm{depth})} \tag{1}$$

   In general, experiments designed to detect this statistical NMR signal use ensembles of shallow NV centers on the order of a few of nanometers. This provides for extremely high magnetic spatial resolution and localizes the detection. Furthermore, due to the small dominant sensing volume resulting from the shallow NV layer ($r \sim 2\times$ avg. depth), the statistical polarization is very high and dominates over potential thermal NMR signals (Supplementary Figure 2), making it the ideal regime to apply this technique. Signals of this origin have been detected with pulse sequences designed for variance detection, using both photodiodes and cameras as detectors[1–3]. Such measurements have proven invaluable in the study of surface dynamics and small volume samples due to their ease of use and minimal timing requirements, allowing for the use of slow detectors.

2. **Boltzmann NMR signal:** An external magnetic bias field, induces a preferential alignment of the sample spins along the B-field according to a Boltzmann distribution as a consequence of the Zeeman splitting of the spin energy levels. This Boltzmann polarization of the sample can be excited by applying a resonant radio frequency pulse to the sample, inducing a coherent precession around the B-field. The exchange of sample spins thus excited with spins surrounding the dominant detection volume does not affect the coherence time as long as the B-field is sufficiently homogeneous over the diffusion length scale of an experiment or over a sample containing structure. Therefore, the line width of a Boltzmann NMR signal is not limited by diffusion through the dominant detection volume. Instead, the homogeneity of the bias field becomes an extremely important factor in the design of the experiment and is the main limiting factor for our measurements.

For higher proton counts, the Boltzmann NMR signal becomes dominant over the statistical signal, which is why diamonds with NV layers on the order of μm and thus larger detection volumes are typically used for such experiments.

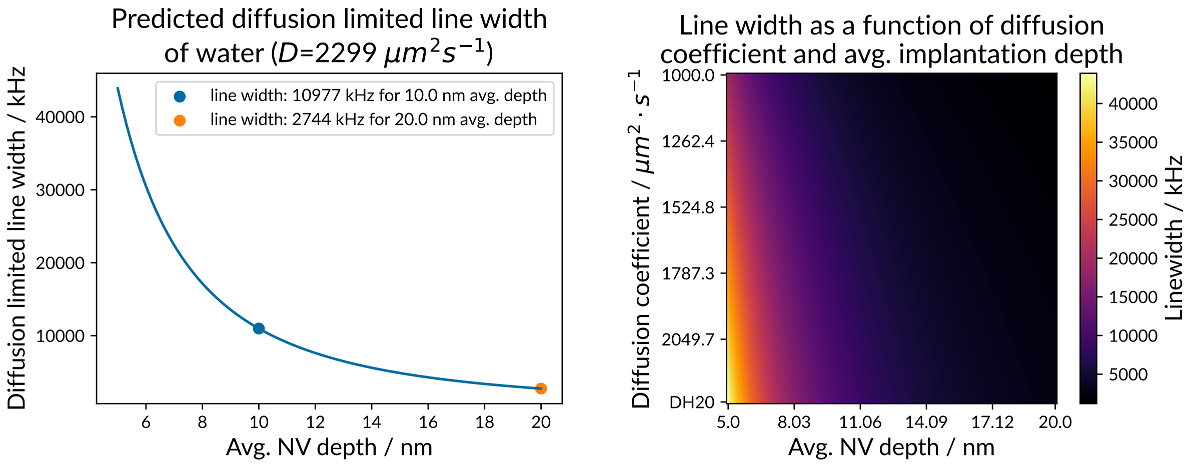

Supplementary Figure 1: Estimated diffusion limited line widths of statistical NMR signals. Left: Estimated diffusion limited line width of a statistical NMR signal based on the average diffusion time through the dominant sensing volume as a function of implantation depth. Right: Color-coded estimated diffusion limited line width as a function of diffusion coefficient and NV implantation depth

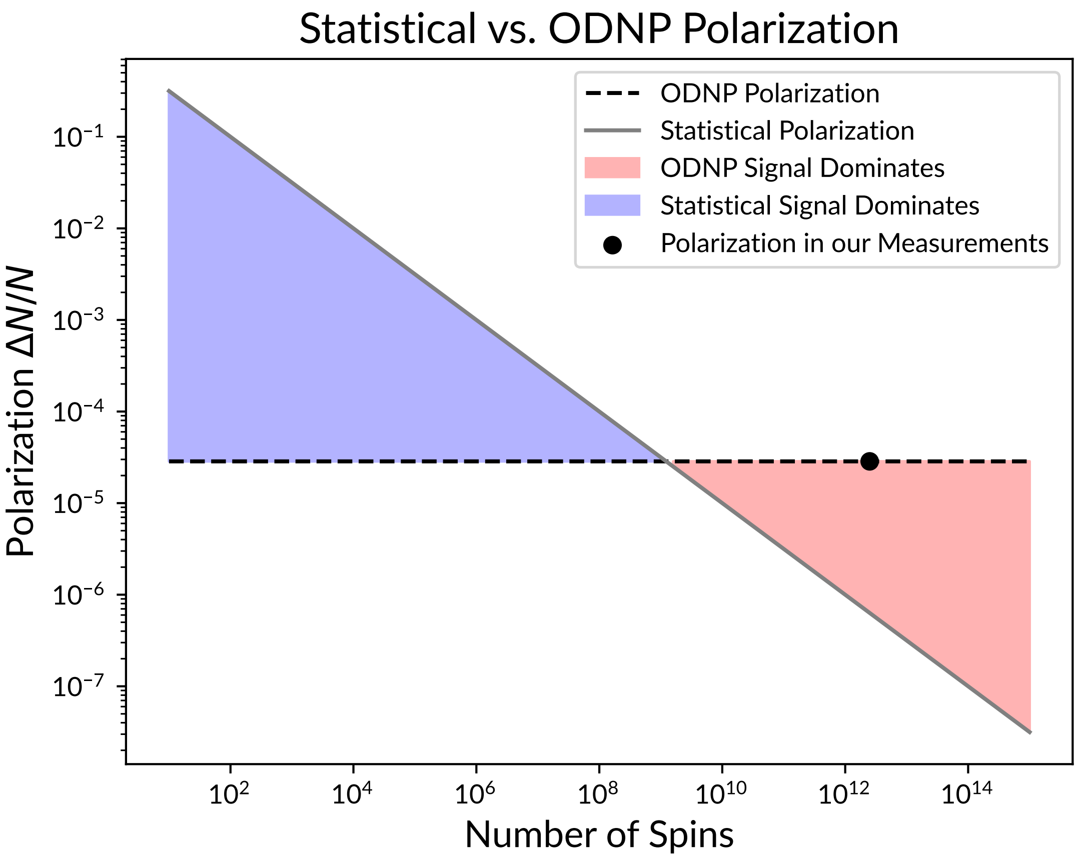

Supplementary Figure 2: Statistical and Boltzmann/ODNP polarization as a function of the number of spins in the detection volume. ODNP values are computed according to our experimental parameters.

Supplementary Note 2

Quantum Sensing Tutorial

For detailed explanation of NV-based quantum sensing, we refer the reader to excellent reviews by Degen et al., Levine et al., and Allert et al.[4–6]. The NV center in diamond is a point defect in the crystal lattice where one of the carbon atoms is replaced by a nitrogen atom, and an adjacent lattice site is left vacant. It possesses three key properties, which form the basis for its quantum sensing applications:

1. Its electronic spin state can be optically initialized.
2. Its electronic spin state can be coherently manipulated with microwave pulses and has long coherence and relaxation times.
3. Its electronic spin state can be read out optically due to its spin state-dependent fluorescence intensity.

NV quantum sensing measurements typically require three steps. First, the system is optically polarized into a defined spin state by laser excitation. Second, it is transformed into the desired

initial sensing state, which evolves under the external perturbation (i.e. magnetic field or NMR signal to be measured). Finally, the resulting spin state, which encodes the measurement, is read out optically, exploiting the spin state-dependent fluorescence. This cycle can be repeated many times to increase the SNR.

NV-ESR experiment and magnetic field alignment

The electron spin resonance (ESR) experiment allows us to find the NV-resonance frequency (also called optical detected magnetic resonance (ODMR)). Its electronic ground state is a triplet state, situated within the diamond band gap. The corresponding magnetic sublevels $m_s = |0\rangle$ and $m_s = \pm|1\rangle$ are energetically separated by a zero-field splitting of $D = 2.87$ GHz, while the degeneracy of the $m_s = \pm|1\rangle$ states is lifted by external magnetic fields B_0 (Zeeman effect). The spin state $m_s = |0\rangle$ is brighter (higher fluorescence intensity) compared to the $m_s = \pm|1\rangle$ states. In our experiments, we apply a magnetic field B_0 (~84 mT) along the NV center's principal axis. After optical polarization of the NV center into the bright $m_s = |0\rangle$ state, the frequency of the applied microwave pulse is swept. At the resonance condition $m_s = |0\rangle \to +|1\rangle$ or $-|1\rangle$, the fluorescence intensity decreases (~5.2 GHz at ~84 mT for the $m_s = |0\rangle \to +|1\rangle$ state).

NV-Rabi experiment

The Rabi experiment allows the determination of the microwave pulse duration, which rotates the spin state by $\pi/2$ or π. These are essential control parameters for most quantum sensing experiments. We sweep the MW pulse duration at the spin resonance frequency determined in the ESR experiment (~5.2 GHz for $|0\rangle \to |+1\rangle$ at ~84 mT), which results in an oscillation of the fluorescence intensity (and the NV center's spin state between $m_s = |0\rangle$ and $|+1\rangle$, Rabi oscillations). The pulse duration resulting in the first intensity minimum rotates the spin state by π from $|0\rangle \to |+1\rangle$ and is thus termed a π pulse. A pulse with half this duration rotates the system into a superposition between $|0\rangle$ and $|+1\rangle$. This pulse is termed a $\pi/2$ pulse.

NV-CASR experiment

The coherently averaged synchronized readout (CASR) experiment is used for high spectral resolution RF measurements[7,8]. After bringing the NV center's spin state into a superposition of $|0\rangle$ and $|+1\rangle$ by applying a $\pi/2$-pulse, it becomes maximally sensitive to changes in the magnetic field. The NV center's spin accumulates a phase ($\Delta\phi$), which can be translated into a change in fluorescence intensity by applying another $\pi/2$ pulse. While a static offset in the magnetic field leads to an increasing phase accumulation, the oscillating nature of an RF or NMR signal will lead to an inefficient phase accumulation. To counteract this effect, we apply a precisely timed train of π pulses (Dynamical Decoupling sequence - DD) at twice the rate of the RF/NMR signal frequency. The π pulse train / DD sequence acts as a rectifying function of the RF signal when applied at its inflection points, causing the NV center spin to accumulate more phase with each RF oscillation. When the π pulses are applied at the maxima and minima of the RF oscillation, they suppress phase accumulation (Supplementary Figure 3). The effectiveness of such DD sequences in enabling phase accumulation is thus highly dependent on the phase of the RF signal relative to the DD sequence. A final $\pi/2$ pulse is applied to map the phase accumulation on the NV's spin state population, which can be read out by the fluorescence intensity. Precisely timed repetition of this measurement sequence leads to a periodically varying efficacy in phase accumulation and, thus, fluorescence intensity,

determined by the difference in repetition rate and RF signal frequency. The fluorescence oscillates over the readouts at an aliased frequency of the RF signal. We term the observed frequency "CASR frequency".

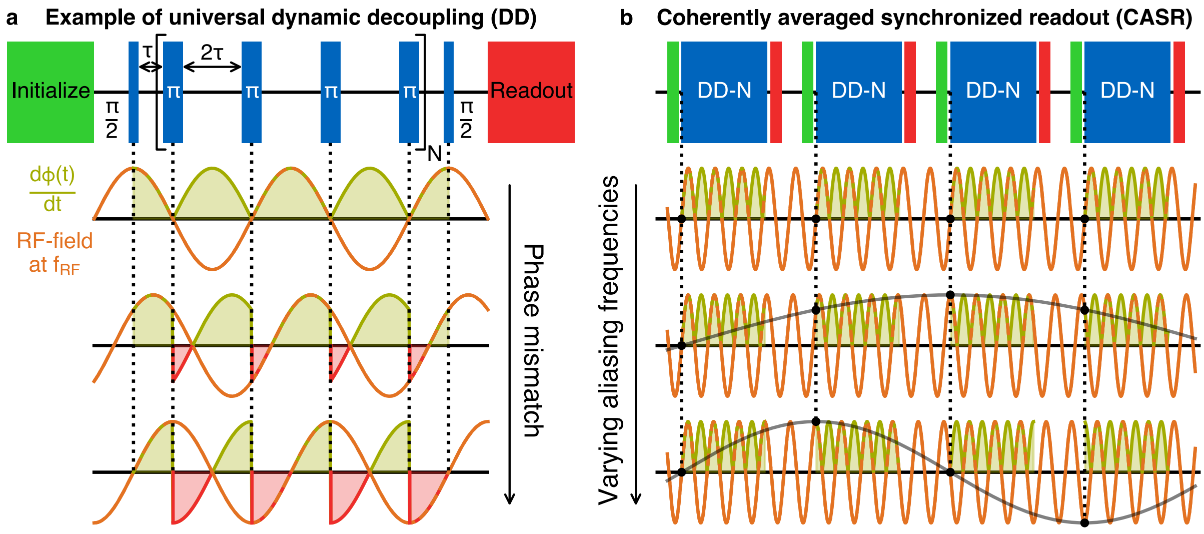

Supplementary Figure 3: Pulsed RF sensing overview. **a**, Dynamical decoupling (DD) with the XY4-N sequence contains laser pulses for initialization (green), readout (red) and microwave pulses (MW, blue) for spin manipulation. The MW pulse sequence consists of an initial $\pi/2$ pulse to bring the system into superposition of $|0\rangle$ and $|1\rangle$. A block of equidistant π pulses (spacing 2τ) is repeated N times. A last $\pi/2$ maps the phase accumulation to the fluorescence intensity of the NV center, which is read out. If the π pulses are placed at the inflection points of the sensed RF signal, then the accumulated phase ($\Delta\phi$) during the measurement is maximal. If the position of the π pulses is mismatched to the phase of the sensed field, the amount of phase accumulation between two π pulses is reduced until it cancels out for a 90° mismatch. **b**, The CASR sequence is based on full DD blocks synchronized to the sensed AC-field (f_{ac}). For matching frequencies, the phase accumulation during each DD sequence has the same value, resulting in an unchanged fluorescence intensity from each DD block to the next. If the frequencies mismatch slightly, the phase of the sensed field changes during the measurement, resulting in a fluorescence oscillation at the aliased frequency of f_{RF} and the repetition frequency of the dynamical decoupling-readout cycle. The frequency resolution of this measurement is only limited by the clocks of the involved technical equipment and is used to detect NMR spectra with Hz resolution.

Supplementary Note 3

Spatial resolution of OMRM

The spatial resolution of our OMRM technique is ultimately limited by the optical diffraction limit. Imaging through the bulk of our diamond sample, which has a thickness of approximately ∼500 µm and a high refractive index, introduces optical aberrations that limit the achievable resolution. As demonstrated by Nishimura et al. (2024), the use of a 0.7NA objective to image a field of view of ∼200 µm through a diamond with a thickness of ∼500 µm imposes an upper limit on the optical resolution of ∼3 µm, which is below our magnetic resolution[9].

The magnetic resolution in our experiment, which we define as the extent to which the signal leaks beyond the microfluidic channel boundary, is determined by the depth of the NV layer. The radius of the dominant sensing volume for a given sensing location is approximately twice the average NV depth[10]. The overall spatial resolution is determined by both the optical and magnetic resolutions. When the NV-layer thickness is below the optical limit, the optical resolution predominates, while for layers thicker than the optical limit, the magnetic resolution becomes the dominant factor. We performed OMRM with a ∼10 µm as well as a ∼40 µm thick NV ensemble. The expected dominant sensing volume radius/resolution, therefore, is $\sim \frac{10\,\mu\text{m}}{2} \cdot 2 \approx 10\,\mu\text{m}$ and $\sim \frac{40\,\mu\text{m}}{2} \cdot 2 \approx 40\,\mu\text{m}$. In Supplementary Figure 4 we observe NMR signal leakage beyond the microfluidic channel boundaries. The extent of this leakage is in good agreement with the expected magnetic resolution. The signal obtained with the ∼ 40 µm NV ensemble leaks approximately ∼ 40 µm beyond the microfluidic channel walls, while the signal leakage is limited to ∼ 10 µm for the associated NV ensemble (Supplementary Figure 4). In these experiments, the NV principal axis is orthogonal to the direction of the microfluidic channel.

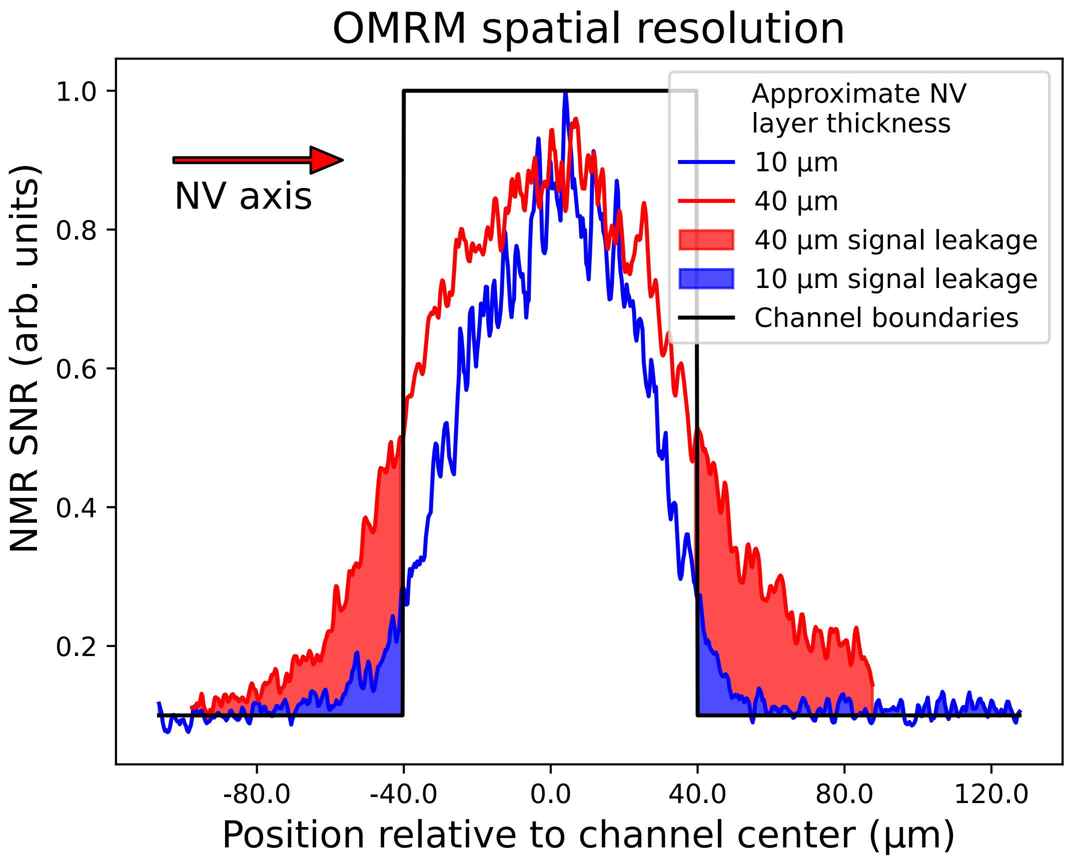

Supplementary Figure 4: Spatial resolution of OMRM. Black: schematic microfluidic channel boundaries. The channel width was ∼ 80 µm. Red: NMR signal-to-noise ratio obtained from OMRM measurements using a ∼ 40 µm NV ensemble. The signal leaks approximately ∼ 40 µm beyond the channel walls. Blue: NMR signal-to-noise ratio obtained using a ∼ 10 µm NV ensemble. The signal leaks approximately ∼ 10 µm beyond the channel.

Supplementary Note 4

Sensitivity

We define the signal-to-noise ratio (SNR) for our FFT spectra as

$$\mathrm{SNR} = \frac{s}{\sigma} \tag{2}$$

where s is the signal amplitude of the spectrum and σ is the standard deviation of the complex-valued FFT spectrum in a signal-free region as calculated by NumPy's std() method on complex-valued arrays. Let $\widehat{}$ denote time and volume normalization. With this, we can rewrite the SNR with measurement time t and sensor volume v as

$$\mathrm{SNR} = \frac{\widehat{s} \cdot t \cdot v}{\widehat{\sigma} \cdot \sqrt{t} \cdot \sqrt{v}} \tag{3}$$

$$\mathrm{SNR} = \frac{\widehat{s}}{\widehat{\sigma}} \cdot \sqrt{t} \cdot \sqrt{v} \tag{4}$$

The SNR by itself, albeit being easy to obtain, does not provide information about the sensitivity η of an experiment in absolute terms. We supply a calibration signal with known magnetic field magnitude and compute the SNR for the resulting peak. We denote the magnetic field amplitude A in Tesla and obtain the magnetic noise floor σ_B of the measurement by:

$$\sigma_B = \frac{A}{\mathrm{SNR}} \tag{5}$$

To compute the sensitivity as the time and volume normalized noise floor, we rewrite Eq. 5 as:

$$\eta = \frac{A}{\widehat{\mathrm{SNR}}} \tag{6}$$

$$\widehat{\mathrm{SNR}} = \frac{\widehat{s}}{\widehat{\sigma}} \tag{7}$$

$$\widehat{\mathrm{SNR}} = \frac{\mathrm{SNR}}{\sqrt{t} \cdot \sqrt{v}} \tag{8}$$

$$\eta = \frac{A}{\frac{\mathrm{SNR}}{\sqrt{t} \cdot \sqrt{v}}} \tag{9}$$

$$\eta = \frac{A}{\mathrm{SNR}} \cdot \sqrt{t} \cdot \sqrt{v} \tag{10}$$

Magnetic sensitivity estimation

We estimate the sensing performance of our measurement using a number of different metrics. The most important measurement parameters are found in Supplementary Table 1.

Supplementary Table 1: Measurement parameters for FPS dependent sensitivity determination

pulse sequence block (µs)	N frames	height / px [a]	width / px	t-measure / s	fps	averages
317.842960	6300	500	704	1.001205	6292.42	1
285.834040	7000	448	704	1.000419	6997.07	1
256.632920	7800	400	704	1.000868	7793.23	1
222.377760	9000	348	704	1.000700	8993.71	1
200.476920	10000	300	704	1.002385	9976.21	1
160.044600	12500	248	704	1.000279	12496.52	1
133.651280	15000	200	704	1.002385	14964.32	1
97.149880	20700	148	704	1.005501	20586.75	1
93.218960	21582	100	704	1.005926	21454.86	1

[*] For each set of parameters, the measurement was repeated three times.

[a] Increasing the field of view along this dimension sets the fps. The analysis region remained the same for all measurements.

Magnetic sensitivity as a function of frame rate

To determine the effect of the camera frame rate (frames per second, FPS) on the time and volume normalized sensitivity, we supply a 10 nT RF calibration signal and sweep the camera frame rate while keeping the measurement time at 1 second. Because our measurement is camera-based, slight inhomogeneities in the laser illumination can lead to differences in sensitivity across the sensor. For our analysis, we pick the edge of the laser spot to gain a conservative estimate. For each measurement associated with a particular frame rate, we Fourier transform and median filter the data as described in Supplementary Note 7 . We select the same 30 neighboring 3×3 pixel regions on the sensor ($1\,\mu\text{m}^2$) for each measured frame rate. Each 3×3 pixel region is averaged before calculating the SNR and the sensitivity. The obtained maximum value and noise floor are depicted in Supplementary Figure 5 while the computed SNR and sensitivity are visualized in Supplementary Figure 6. The varying sampling rate of the RF calibration signal leads to numerical artifacts in the FFT, causing the maximum peak value to vary. Despite the FFT artifacts, we observe an approximate sensitivity range of $10 - 30\,\frac{\text{nT}}{\sqrt{\text{Hz}}} \cdot \sqrt{\mu\text{m}^3}$ which scales according to the frame rate (approx. 22000 to 6000 fps).

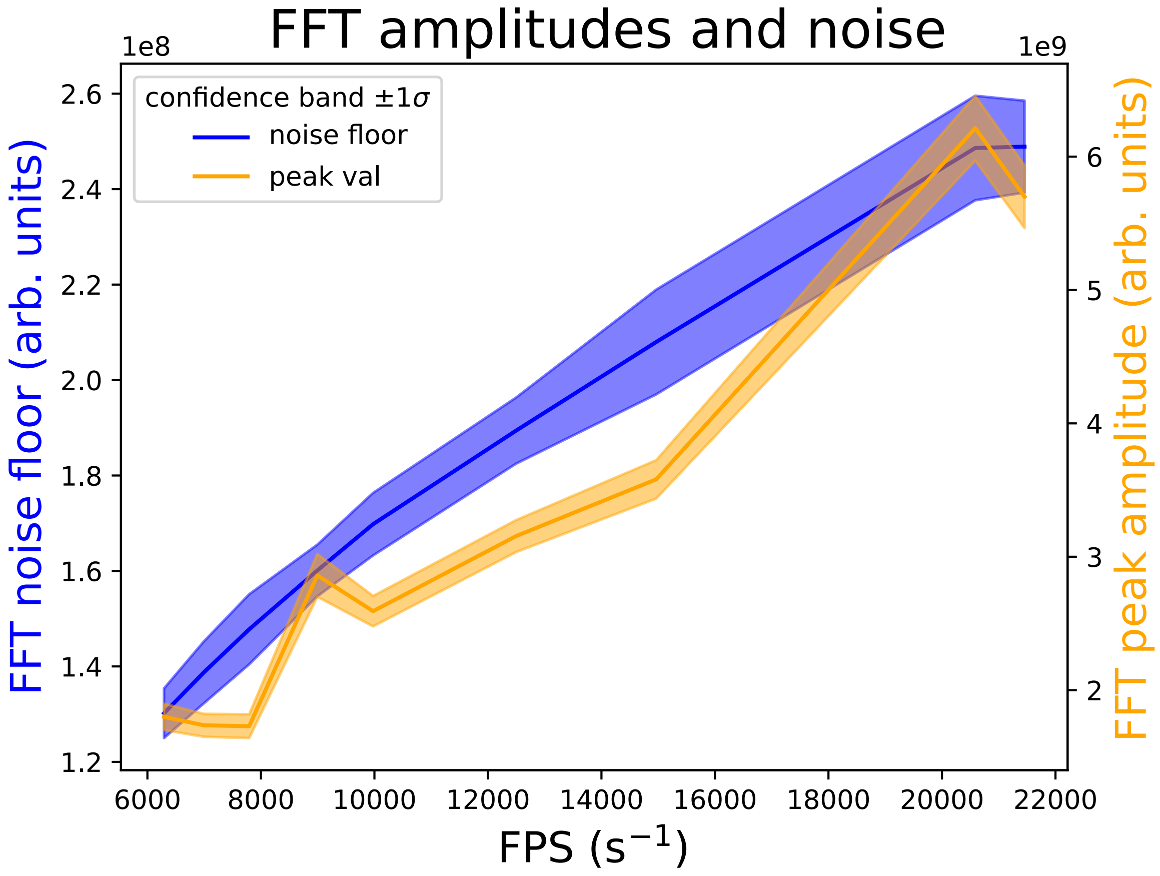

Supplementary Figure 5: Noise and signal amplitudes as a function of FPS. Blue: FFT noise floor. As the number of measurements per second grows, the noise floor grows with the square root of the number of frames. Orange: Calibration signal peak height. The peak height grows approximately linearly as the number of measurements / the frame rate increases. Due to numerical artifacts of the FFT for different sampling rates, the amplitude fluctuates strongly.

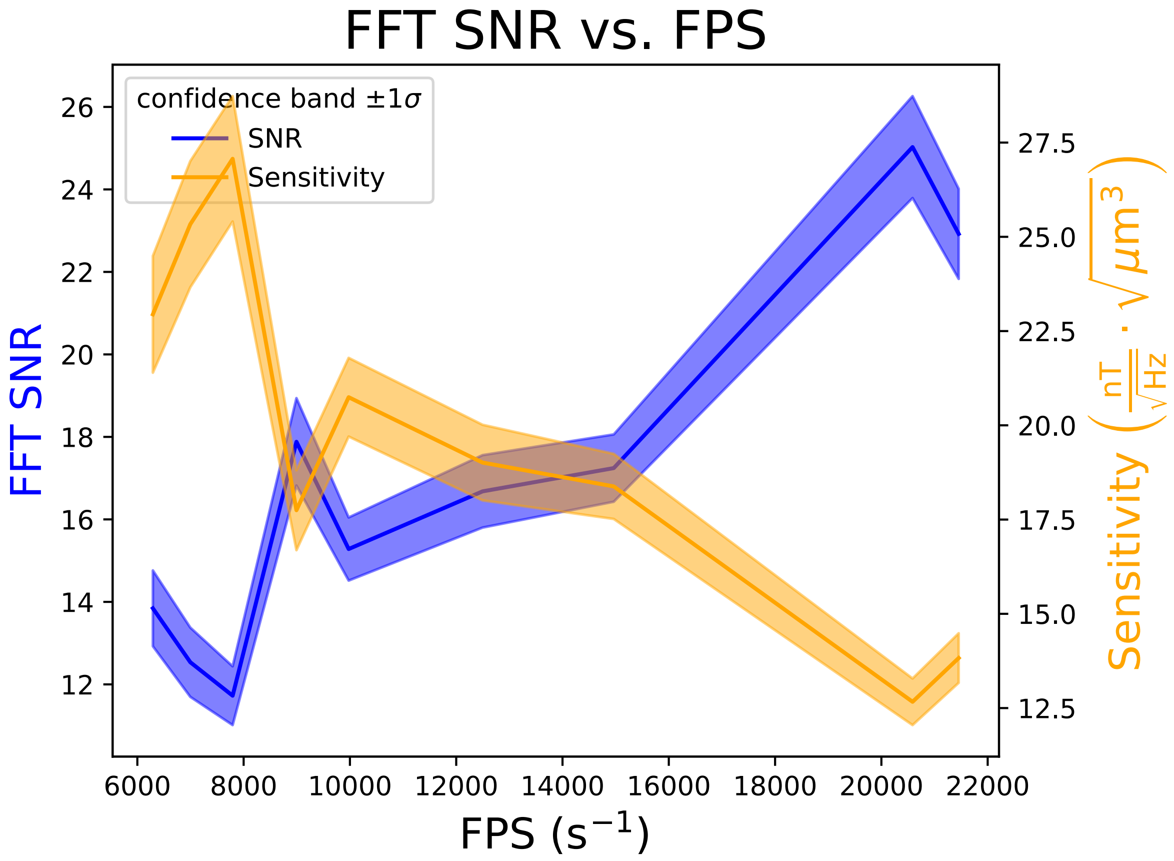

Supplementary Figure 6: SNR and sensitivity as a function of FPS. Blue: FFT SNR as a function of frame rate. Orange: Estimated time and volume normalized sensitivity. Due to numerical artifacts of the FFT for different sampling rates, the amplitude fluctuates strongly.

Magnetic sensitivity as a function of averaged pixels

We characterize the sensor performance for a fixed frame rate of ∼6300 fps. The dataset is loaded, Fourier transformed, and median filtered. We then partition the sensor into 40 50×50 pixel regions. For each pixel region, we increase the observed subregion from 1×1 to 50×50 pixels and compute the noise floor and the SNR for averaged pixels. The result is depicted in Supplementary Figure 7. Because the sampling rate is constant in this analysis, there are no FFT artifacts. Both the noise floor and the SNR scale with expected square root dependence on the number of pixels.

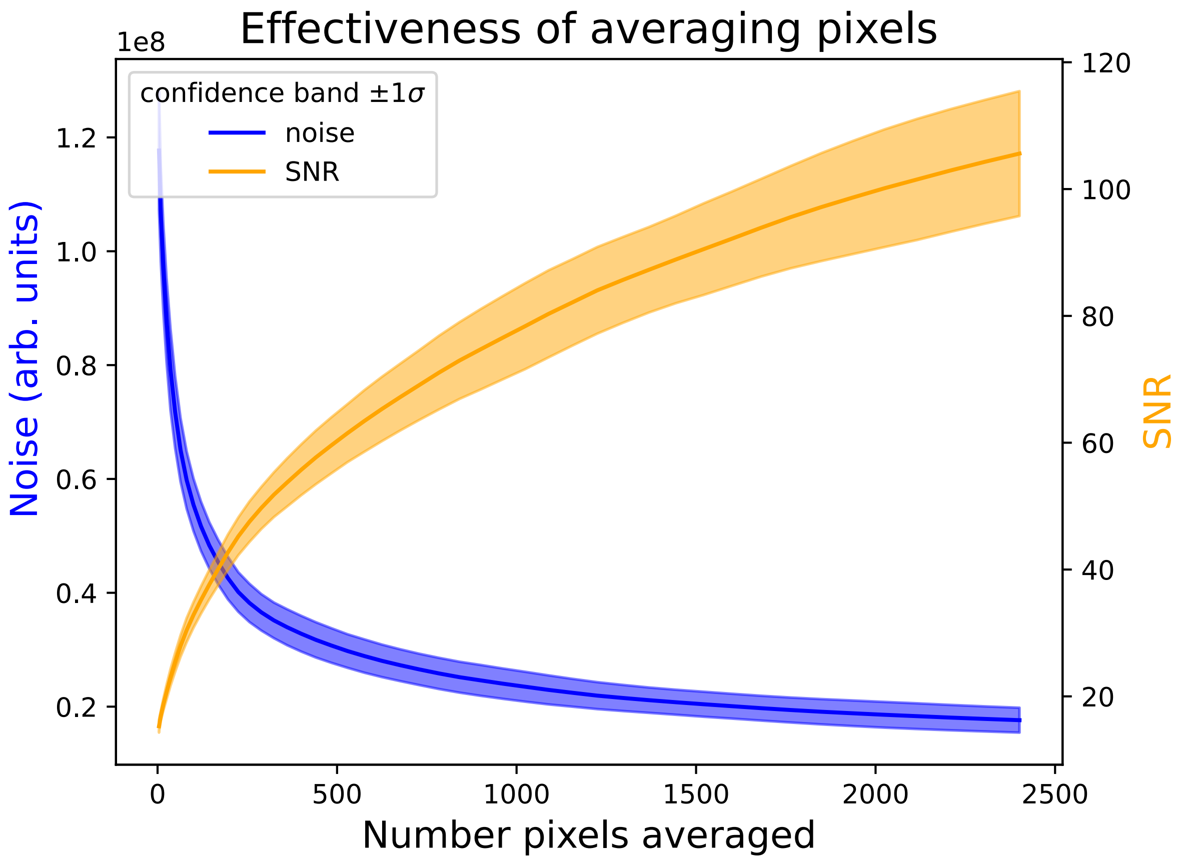

Supplementary Figure 7: Noise and SNR as a function of averaged pixels. Blue: FFT noise floor. Orange: Calibration signal SNR.

Magnetic sensitivity as a function of averages

We characterize the sensor performance for a fixed frame rate of 6000 fps. 189 CASR calibration measurements with 100 averages each were recorded. The individual datasets are loaded, Fourier transformed and analyzed in $(10\,\mu\text{m})^3$ chunks (30×30 px). Each successive dataset is added onto the one before to analyze the effect of progressively longer averages. In the limit of one long averaging measurement, as opposed to many short chunks, filtering can not be applied while accumulating the measurements. Because of this, no 2D median filter was applied in this analysis. The results are visualized in Supplementary Figure 8. Because the sampling rate is constant in this analysis, there are no FFT artifacts.

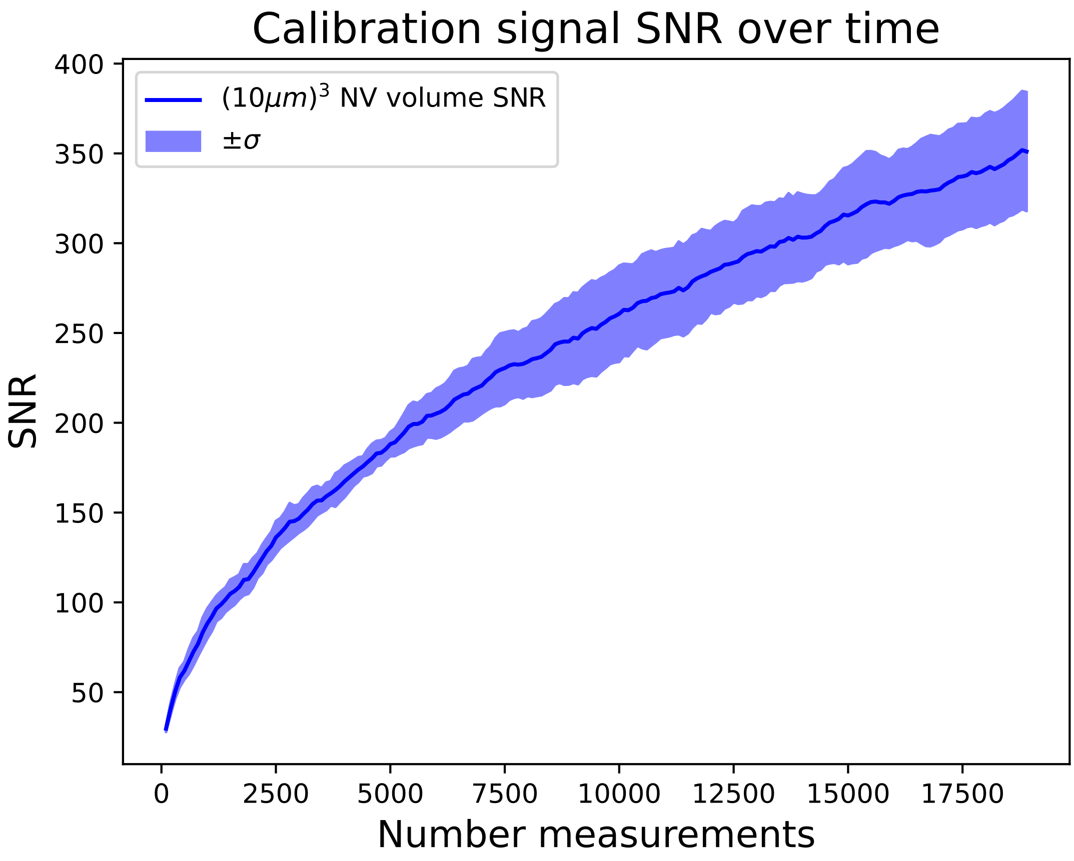

Supplementary Figure 8: SNR as a function of averaged measurements. Averaging progressively more calibration signal measurements retains the expected square root scaling of the SNR.

Supplementary Note 5

Camera considerations

Selecting an appropriate camera for a quantum sensing experiment is a complex undertaking, requiring careful consideration of various experimental parameters. In this section, we will outline a step-by-step approach to determine the right choice of camera for our OMRM experiment (See Supplementary Figure 9). First, the required magnetic spatial resolution must be determined as a function of the sample in question. The thickness of the NV layer must be chosen appropriately, as this is the primary determining factor of the magnetic spatial resolution. At this point, the NV doping parameters can be adjusted to better match the diamond sensor to the application, which will influence the brightness and the coherence times. Together, these parameters determine the amount of signal-bearing fluorescence from the diamond per volume and per readout cycle. It is important to note that the amount of light that can effectively be used for readout is not a function of laser power, but rather depends entirely on the characteristics of the diamond. Given the selected magnetic resolution and the desired optical resolution, the next step is to select the appropriate optics. The choice of magnification, light collection efficiency, and field of view determine the spread and distribution of the fluorescent

signal across the pixels. Finally, the camera must be selected to best match the constraints imposed by the previous choice of diamond and optics. Importantly, the pixel size (pixel pitch) should be matched to the chosen magnification and resolution of the optics in the image plane. In order to avoid limiting the resolution, oversampling is generally preferred. The full well capacity of the sensor is largely determined by the pixel size. Depending on how well the diamond, optics, and camera sensor are matched, the quantum efficiency of the sensor should be considered. Since the quantum efficiency typically ranges from $\sim 30\%$ to $\sim 90\%$, it affects the sensitivity of a measurement by a factor of up to $\sim \sqrt{3}$. Finally, the duty cycle of the experiment deserves consideration. The upper limit of the duty cycle in the case of dynamical decoupling sequences is imposed by the coherence time of the NV centers, which is typically in the range of ∼10-100 microseconds. It is advantageous to minimize the dead time between measurements. For example, for an experiment with a duty cycle of 100 µs, going from a very slow camera with a frame rate of 10 fps to a very fast camera with a frame rate of 10 000 fps, would increase the sensitivity by a factor of $\sim \sqrt{1000}$. Furthermore, the limited lifetime of many physical phenomena of interest, such as that of an NMR signal, requires that the camera to be able to take an adequate number of measurements for each observation or risk extreme averaging times.

In summary: It is recommended to select the diamond and optical components in accordance with the specific requirements of the experiment, and then match the camera to these boundary conditions, rather than adapting the experiment to the camera's parameters. The choice of diamond and optics imposes strict constraints on the camera specifications. Once a rough match of the camera to these constraints has been achieved, the major difference between cameras lies in their achievable frame rates. Optimizing the frame rate can result in substantial increases, potentially on the order of $\sim \sqrt{1000}$. However, it is important to note that this matching process is extremely challenging, and we have not achieved a perfect match in our reported work. Despite our best efforts, some deviations impacting readout quality remain (Supplementary Note 6), underscoring the difficulty of fully aligning the camera parameters with the experimental requirements (e.g. the diamond properties).

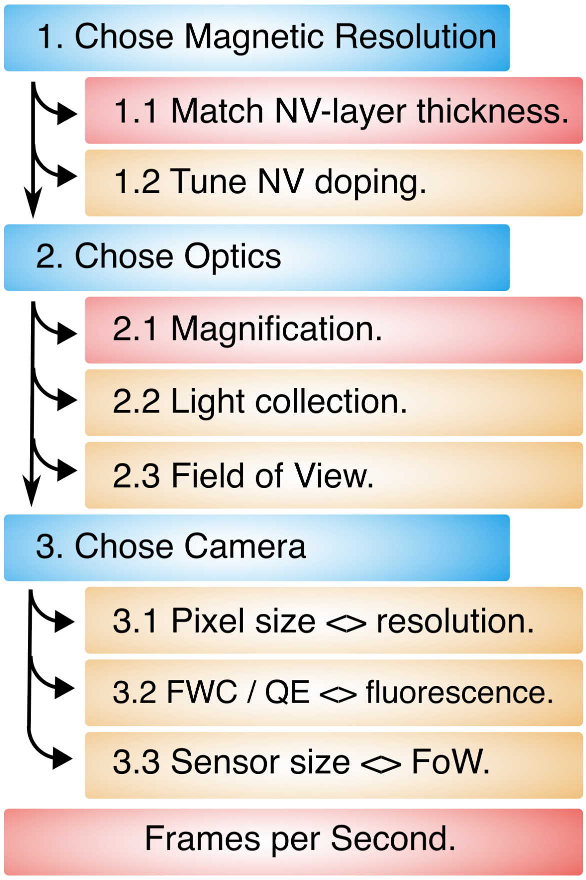

Supplementary Figure 9: Camera selection process

We selected different cameras for our OMRM technique based parameters such as pixel size, quantum efficiency, readout characteristics and so forth. We evaluated the CASR magnetic field sensitivity using the camera listed in Supplementary Table 2. When analyzing the calibration signal sensitivity, we found two parameters to be most important in agreement with the discussion above.

1. The camera frame rate.

2. Whether the camera can stream data / data transfer speeds.

The most determining factor for sensitivity in our experiment were the number of frames we could continually read out over a sustained period of time (multiple hours) at high frame rates. While there are many high-speed cameras on the market, most of them accumulate frames in local storage. Once storage is full, the images need to be transferred to the computer for averaging and analysis, limiting the time for uninterrupted averaging. Thus, the better metric for evaluating camera performance is the effective frame rate, i.e., the frame rate taken as an average of a period of at least one hour. This is best provided by high-speed streaming cameras, such as the EoSens1.1MCX12-CM, we used in our experiments (See Supplementary Table 3). We would like to note that this camera is still not ideal for our applications (e.g. FPS and quantum efficiency (Supplementary Note 10)).

Supplementary Table 2: Comparison of selected camera models

Company	Model	Effective FPS[a]/ s^{-1}	N pixel	streaming
SVS-Vistek	EoSens1.1MCX12-CM	3674	1.1e6	yes
Heliotis	Helicam C3	52[b]	90e3	no
Basler	ace acA1920-155um	164	2.3e6	yes

[a] Frames per second at full resolution.
[b] This lock-in camera has an internal demodulation frame rate of 1e6 FPS. It can record finished images at 3800 fps. The camera can store a maximum of 512 frames in the camera buffer, which take ~10 seconds to transfer to a computer. This reduces the effective FPS to 52 when recording over multiple hours.

Supplementary Table 3: EoSense1.1MCX12-CM parameters

FPS[a]	Sensor	Pixel size	FWC[b]	QE[c]	Resolution	Color
3674	LUX13HS	13.7x13.7 μm^2	20,000	$\leq$ 30%	1.1 MP	Mono

[a] Frames per second at full resolution.
[b] Full Well Capacity.
[c] Quantum Efficiency.

Supplementary Note 6

Sensor response

Supplementary Figure 10 visualizes the complex interplay between laser power distribution and camera sensor saturation for our CASR measurements of the calibration signal. The saturation of our camera sensor is in a suboptimal regime in our measurements (Supplementary Note 5). We observe that this sometimes leads the camera sensor to generate disproportionately high noise in regions of very low light exposure. Simultaneously, regions with higher camera saturation produce a disproportionately low signal and noise. This results in an overall flat Signal to Noise Ratio across the sensor.

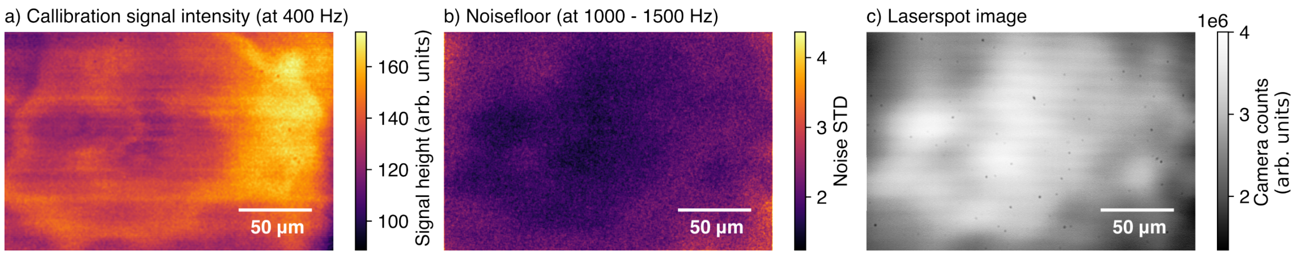

Supplementary Figure 10: Calibration signal image components. **a**, effective CASR calibration signal response. **b**, FFT noise floor of CASR calibration signal. **c**, laser spot illuminating the measurement area.

Supplementary Note 7

Data Analysis

Frequency correction

To mitigate the effects of slow magnetic field drifts caused by temperature variations in the laboratory during averaging, we save snapshots of the raw measurement data every ∼5 minutes. Taking the mean over multiple pixels increases the SNR enough to track the NMR signal frequency between snapshots taken every ∼5 minutes during the measurement. After extracting the NMR resonance frequency for each snapshot, we fit a polynomial of sufficiently high order to heuristically describe the frequency drift (7th order for the straight and diagonal channels - left and center in Fig. 4; 11th order for T-shaped channel - right in Fig. 4). Before applying the frequency correction, we detrend the real-valued time domain signal using a 5th order polynomial fit and approximate the analytic signal by Hilbert transforming it using `scipy.signal.hilbert`. The resulting analytic/complex signal can be frequency corrected by multiplying it with $e^{i\Delta ft}$, where Δf is obtained from our heuristic fit. The frequency-corrected snapshots are added up to effectively average the measurement over multiple hours.

Filtering

We Fourier transform the obtained per pixel NMR signal to obtain the per pixel NMR spectrum. This results in a sequence of images where the pixels in each image encodes the FFT amplitude for a specific frequency bin rather than the fluorescence intensity. We take advantage of the fact that oversampled neighboring pixels should not contain strongly divergent peak amplitudes for any frequency bin in the FFT spectrum, by applying a 2D median filter for each frequency bin image. Note that this filter is applied to the imaginary and real parts of the image separately. For the data analysis in the main text, we used `scipy.ndimage.median_filter`. However, for supplementary information analyses, using this filter was prohibitively expensive, so we used a custom build high performance 2D median filter written in Rust.

We evaluated the obtained spectra both by fitting a Lorentzian model function and by analyzing the raw numeric data. In both cases, we calculated the per-pixel SNR by observing the ratio of the maximum absolute value divided by the standard deviation of the complex-valued FFT noise floor. For any analysis other than the signal amplitude, all data points with an SNR above a certain threshold were retained while the others were skipped in the analysis (Fig. 4 from left to right SNR > 8, 3, 5). We term this the SNR mask.

Fitting

In most NMR applications the complex valued spectrum, obtained from Fourier transformation of the time domain signal, is phase corrected. By retaining the absorption mode only, a simplified Lorentzian fitting function, containing only the real part may be used. Because of the inherit difficulty of phase correcting ∼ 300-thousand complex valued NMR spectra, we instead fit the full complex value Lorentzian obtained by Fourier transforming an exponentially decaying signal. See Supplementary Note 7 lower half for more details and derivations.

Our Fourier-transformed signal is most accurately described by the following Lorentzian function:

$$F(f, S_0, \Gamma, f_0, \phi) = S_0 \cdot e^{i\phi} \cdot \frac{\Gamma - i(f - f_0)}{\Gamma^2 + (f - f_0)^2} \tag{11}$$

$$\text{where} \tag{12}$$

$$f = \text{frequency} \tag{13}$$

$$f_0 = \text{center frequency} \tag{14}$$

$$\Gamma = \text{line width} \tag{15}$$

$$\phi = \text{phase} \tag{16}$$

$$S_0 = \text{amplitude} \tag{17}$$

$$\tag{18}$$

This matches the sign of the Fourier transform exponential function as it is implemented in SciPy's `fft` and will thus lead to a proper fit (See Supplementary Note 7 lower half).

We rewrite the Amplitude of the Signal as $S_0 = S_\Gamma \cdot \Gamma$ and term the resulting equation the scaled Lorentzian[11]. Rewriting the amplitude term like this sets the maximum peak value to S_Γ, making it the primary value for obtaining the peak height (See Supplementary Figure 11). For a given line width, this makes the peaks directly comparable, and stabilizes the fit against extreme cases.

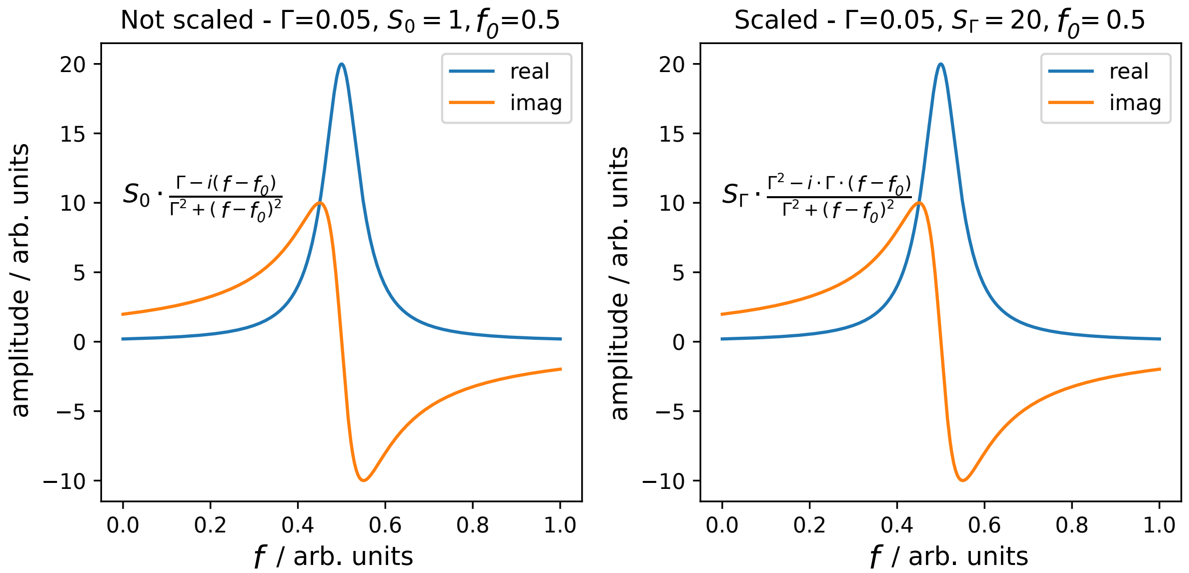

Supplementary Figure 11: Non-scaled and scaled Lorentzian. Left: Lorentzian without scaling. S_0 is directly proportional to the integral over the real part. Right: Scaled Lorentzian. S_Γ is equal to the peak height. The product of $S_\Gamma \cdot \Gamma$ recovers S_0 and thus the integral.

Because the scaled Lorentzian provides better intuition, retains all quantitative information and makes fitting more robust, we use this model for our data analysis. Before visualizing in Fig. 4, we reconstruct S_0 by multiplying the optimized fit parameters $S_\Gamma \cdot \Gamma$ and dividing the resulting value by the signal amplitude of the calibration signal of each per pixel spectrum to minimize the effect of varying laser intensities and MW driving field inhomogeneities.

We fit Eq. 11 for each per pixel complex valued NMR spectrum against an x-axis arbitrarily scaled from 0 to 1 and later adjust the value of the fitted parameters. The optimized parameters are written into an output array at the same index as the associated spectrum. These yield the fit parameter images presented in Fig. 4. We provide an initial guess for the peak location as well as the peak height by extracting the `argmax` or `max` values respectively from the array of absolute values for each spectrum. The initial line width and phase are set to 0.01 and 0 respectively.

Post-correction of fit parameters

We deploy SciPy's `curve_fit` using an unbounded, unconstrained Levenberg-Marquardt optimizer. Because of this, the optimizer may find integer multiples of N for the phase $\phi + N \cdot 2\pi$. It may also simultaneously invert the sign of the amplitude and shift the phase by π. To correct for these phase shifts, we take the absolute value of the amplitude and add a phase of π for each index at which the amplitude was negative. To prevent integer multiples of the phase from complicating the color scale, we take each value of the phase image modulo 2π.

Since we are imaging channel structures in a microfluidic chip, pixels imaging areas outside the channel should carry only minimal NMR signal. Fitting model functions to pure noise tends to result in arbitrary fit parameters that happen to fall into a small local minimum or cause the fit to fail. To mitigate unrealistic fitting results from raw noise spectra, we only consider pixels below the SNR mask for parameters other than S_0.

Numerical analysis

In addition, we analyze the Fourier transformed and median filtered data numerically. The NMR peak amplitude and signal frequency are obtained as the `max` and `argmax` value of the amplitude spectrum, respectively, over the general region of the NMR signal to exclude the calibration signal. The obtained NMR signal frequencies are significantly more coarse-grained than the ones obtained by fitting. We extract the signal phase by calculating the `arctan2` of the ratio of the imaginary and real parts of the signal peak (summed over the immediate range of the peak) according to Eq. 19. Finally, the per-pixel peak signal amplitudes are referenced with the peak amplitudes of the calibration signal to minimize their dependence on the laser intensity and MW driving field homogeneity. The results of this analysis are shown in Supplementary Figure 12. The resulting images closely resemble the images obtained through fitting.

$$\phi = \mathrm{arctan2}(\mathfrak{Im}(S_p)/\mathfrak{Re}(S_p)) \tag{19}$$

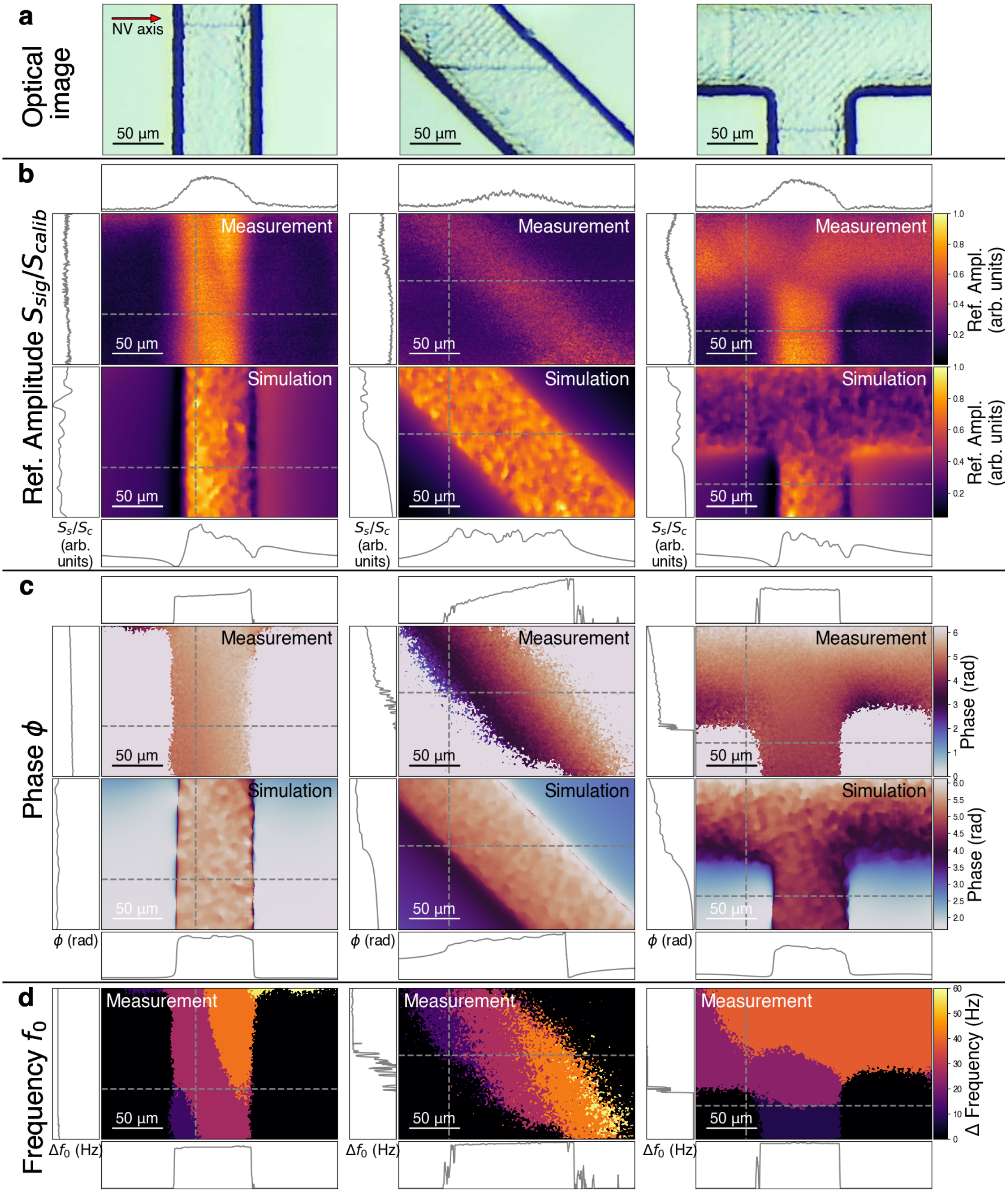

Supplementary Figure 12: Numerically analysed version of Fig. 4. NMR Signal amplitude referenced to the calibration signal amplitude.

Signal Model and Fourier Transform

When fitting a complex Lorentzian to measured data, care must be taken to match conventions pertaining to the Fourier transform to choose the correct model function. We will fully derive the possible model functions and select the correct one for our analysis. (Please note that for

simplicity we drop all constants like 2π etc.)

We model the NMR signal with some slight modifications in accordance with Keeler[12] as:

$$\begin{aligned}
S(t) &= S_0 \cdot e^{if_0 t} \cdot e^{-\Gamma|t|} \cdot e^{i\phi} \cdot \Theta(t) && (20)\\
&\text{where} \\
S_0 &= \text{initial amplitude} && (21)\\
e^{if_0 t} &= \text{complex signal wave} && (22)\\
e^{-\Gamma|t|} &= \text{signal decay} && (23)\\
e^{i\phi} &= \text{signal phase} && (24)\\
\Theta(t) &= \text{Heaviside function} && (25)
\end{aligned}$$

We thus have a decaying complex signal of arbitrary phase that starts at time $t = 0$. The frequency spectrum of this signal can be computed using the standard Fourier transform:

$$\begin{aligned}
F_t[S(t)](f) &= \int_{-\infty}^{\infty} \left[S_0 \cdot e^{if_0 t} \cdot e^{-\Gamma|t|} \cdot e^{i\phi} \cdot \Theta(t)\right] \cdot e^{ift}\,\mathrm{d}t \\
F_t[S(t)](f) &= S_0 \cdot e^{i\phi} \cdot \int_{-\infty}^{0} \left[e^{if_0 t} \cdot e^{-\Gamma|t|} \cdot \Theta(t)\right] \cdot e^{ift}\,\mathrm{d}t \\
&+ S_0 \cdot e^{i\phi} \cdot \int_{0}^{\infty} \left[e^{if_0 t} \cdot e^{-\Gamma|t|} \cdot \Theta(t)\right] \cdot e^{ift}\,\mathrm{d}t \\
&= S_0 \cdot e^{i\phi} \cdot \int_{0}^{\infty} \left[e^{if_0 t} \cdot e^{-\Gamma|t|}\right] \cdot e^{ift}\,\mathrm{d}t && (26)
\end{aligned}$$

Under some conditions, the above Fourier transform can be solved using the following expression:

$$\int_0^{\infty} e^{Ax}\,\mathrm{d}t = \left[\frac{e^{Ax}}{A}\right]_0^{\infty} \qquad (27)$$

For $Re(A) < 0$ (which is the case for all decaying / physical NMR signal) the upper bound converges to 0. The integral thus becomes:

$$\begin{aligned}
\int_0^{\infty} e^{Ax}\,\mathrm{d}t &= \frac{e^{A\cdot\infty}}{A} - \frac{e^{A\cdot 0}}{A} && (28)\\
&= -\frac{1}{A} && (29)
\end{aligned}$$

With this, the Fourier transform from Eq. 26 can be written as:

$$
\begin{aligned}
& F_t[S(t)](f) = && (30)\\
= \quad & S_0 \cdot e^{i\phi} \cdot \int_0^\infty e^{if_0 t - \Gamma t + ift}\,\mathrm{d}t && (31)\\
= \quad & S_0 \cdot e^{i\phi} \cdot \int_0^\infty e^{t\cdot[-\Gamma + i(f_0+f)]}\,\mathrm{d}t && (32)\\
= \quad & S_0 \cdot e^{i\phi} \cdot (-1) \cdot \frac{1}{-\Gamma + i(f_0+f)} && (33)\\
= \quad & S_0 \cdot e^{i\phi} \cdot (-1) \cdot \frac{1}{-\Gamma + i(f_0+f)} \cdot \frac{-\Gamma - i(f_0+f)}{-\Gamma - i(f_0+f)} && (34)\\
= \quad & S_0 \cdot e^{i\phi} \cdot \frac{\Gamma + i(f_0+f)}{\Gamma^2 + (f_0+f)^2} && (35)
\end{aligned}
$$

For the opposite sign in the signal oscillation, this becomes:

$$
\begin{aligned}
& F_t[S(t)](f) = && (36)\\
= \quad & S_0 \cdot e^{i\phi} \cdot \int_0^\infty e^{-if_0 t - \Gamma t + ift}\,\mathrm{d}t && (37)\\
= \quad & S_0 \cdot e^{i\phi} \cdot \int_0^\infty e^{t\cdot[-\Gamma + i(-f_0+f)]}\,\mathrm{d}t && (38)\\
= \quad & S_0 \cdot e^{i\phi} \cdot (-1) \cdot \frac{1}{-\Gamma + i(f-f_0)} && (39)\\
= \quad & S_0 \cdot e^{i\phi} \cdot (-1) \cdot \frac{1}{-\Gamma + i(f-f_0)} \cdot \frac{-\Gamma - i(f-f_0)}{-\Gamma - i(f-f_0)} && (40)\\
= \quad & S_0 \cdot e^{i\phi} \cdot \frac{\Gamma + i(f-f_0)}{\Gamma^2 + (f-f_0)^2} && (41)
\end{aligned}
$$

These are the two Lorentzian peaks we might expect from our Fourier transformed signal.

One point of friction that might occur when trying to fit this model to the frequency spectrum of a recorded signal stems from the fact that there are different conventions for the sign of the integral kernel / test frequency in the Fourier transform. To elucidate this, we will repeat the above derivation for the inverse sign Fourier transform.

$$
e^{ift} \quad \Rightarrow \quad e^{-ift} \tag{42}
$$

Thus, we get:

$$
\begin{aligned}
& F_t[S(t)](f) = && (43)\\
= \;& S_0 \cdot e^{i\phi} \cdot \int_0^\infty e^{if_0 t - \Gamma t - ift}\, \mathrm{d}t && (44)\\
= \;& S_0 \cdot e^{i\phi} \cdot \int_0^\infty e^{t\cdot[-\Gamma + i(f_0 - f)]}\, \mathrm{d}t && (45)\\
= \;& S_0 \cdot e^{i\phi} \cdot (-1) \cdot \frac{1}{-\Gamma + i(f_0 - f)} && (46)\\
= \;& S_0 \cdot e^{i\phi} \cdot (-1) \cdot \frac{1}{-\Gamma + i(f_0 - f)} \cdot \frac{-\Gamma - i(f_0 - f)}{-\Gamma - i(f_0 - f)} && (47)\\
= \;& S_0 \cdot e^{i\phi} \cdot \frac{\Gamma - i(f - f_0)}{\Gamma^2 + (f - f_0)^2} && (48)
\end{aligned}
$$

and

$$
\begin{aligned}
& F_t[S(t)](f) = && (49)\\
= \;& S_0 \cdot e^{i\phi} \cdot \int_0^\infty e^{-if_0 t - \Gamma t - ift}\, \mathrm{d}t && (50)\\
= \;& S_0 \cdot e^{i\phi} \cdot \int_0^\infty e^{t\cdot[-\Gamma - i(f_0 + f)]}\, \mathrm{d}t && (51)
\end{aligned}
$$

$$
\begin{aligned}
= \;& S_0 \cdot e^{i\phi} \cdot (-1) \cdot \frac{1}{-\Gamma - i(f_0 + f)} && (52)\\
= \;& S_0 \cdot e^{i\phi} \cdot (-1) \cdot \frac{1}{-\Gamma - i(f_0 + f)} \cdot \frac{-\Gamma + i(f_0 + f)}{-\Gamma + i(f_0 + f)} && (53)\\
= \;& S_0 \cdot e^{i\phi} \cdot \frac{\Gamma - i(f + f_0)}{\Gamma^2 + (f + f_0)^2} && (54)
\end{aligned}
$$

For a given sign of the integral kernel / Fourier test function, switching the sign of the signal changes the location of the Lorentzian peak between the positive and negative frequency axes. With a positive sign in the integral kernel, the dispersive portion of the peak on the positive axis will dip first before peaking.

If the sign of the integral kernel is reversed, we still find a peak for each of the positive and negative frequencies. In this case, however, the dispersive part of the Lorentzian peaks first and then dips. All derived Lorentzian model functions are visualized in Fig. Supplementary Figure 13.

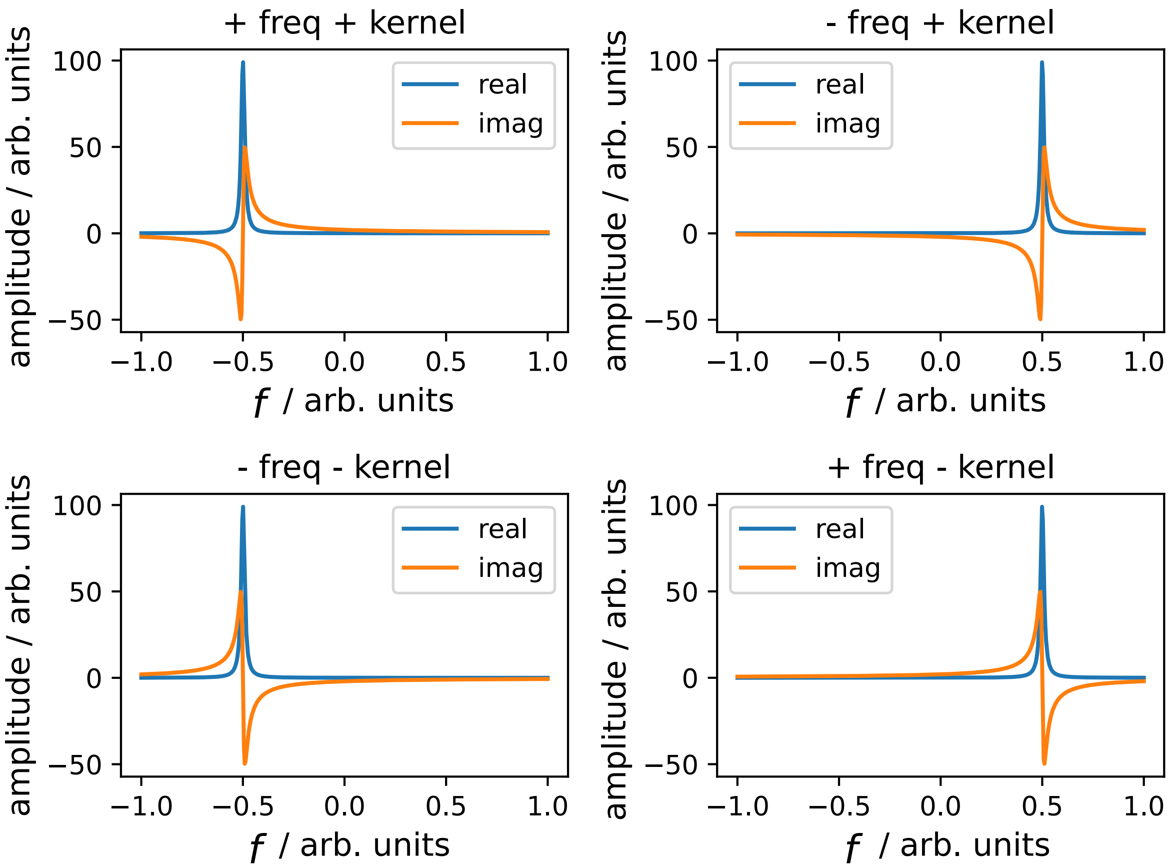

Supplementary Figure 13: Possible complex valued Lorentzian model functions. All four Lorentzian model functions derived above.

It is important to match the fitting model function to the convention used in the DFT/FFT used to process the data. Otherwise, the fit will not converge properly.

OMRM cross-sections and image alignment

The SNR cross-section in Figure 3g is obtained by averaging 11 lines in the lower quarter of Figure 3f. The schematic channel outline is reconstructed by aligning two horizontal lines with an 80 µm spacing to the edges of the signal channel image.
Each column in Figure 4 contains images that have been acquired by wide field optical microscopy, simulation or our OMRM technique. Lining up the regions from the optical microscopy and the simulation to the area recorded on the OMRM microscope is done by eye to obtain qualitative agreement.

Supplementary Note 8

Simulation of the NMR signal

For the simulation of the expected per-pixel NMR amplitude and phase, we model the NV - ^{1}H interaction as a classical dipole-dipole interaction[10].

$$H = -\frac{\mu_0}{4\pi|r|^3}\left[3(\mathbf{m}_1 \cdot \hat{\mathbf{r}})(\hat{\mathbf{r}} \cdot \mathbf{m}_2) - \mathbf{m}_1 \cdot \mathbf{m}_2\right] \tag{55}$$

where $\mathbf{m}_1$ and $\mathbf{m}_2$ encode the ^{1}H dipole moment and the NV center's principal axis respectively. $\hat{\mathbf{r}}$ encodes the normalized distance vector between the two.

We use the microfluidic channel structure from a standard tessellation language (STL) file. To simulate the NV ensemble layer, we generate a 2D array of vectors at a specified distance from the lower face of the microfluidic chip. These vectors represent the NV center's spin ($\mathbf{m}_2$) at each "pixel" of the simulation. The values of each vector are chosen to reflect the orientation of the NV center's principal axis relative to the microfluidic chip. We draw random ^{1}H locations inside the microfluidic chip's volume and check whether it is inside the channel or the chip's volume by counting the number of triangle intersections along a specified axis/ray. We use a modified version of the Möller-Trumbore algorithm to remove back face culling[13]. Once a valid ^{1}H position has been found, the dipole-dipole interaction energy (Eq. 55) is computed for every $\mathbf{m}_2$ in the NV array. For each new valid ^{1}H the interaction energies for each NV center are summed up.

New ^{1}H positions are drawn until the channels have been populated with a pre-specified number of dipoles (50,000 in our case). This process is repeated multiple times (21 in our case) while rotating the ^{1}H orientation incrementally up to 2π around the NV center's principal axis, while keeping it perpendicular to said axis to simulate the precession of the sample spins around the B_0 field.

Finally, a cosine is fitted to the simulated interaction energy values for each "pixel's" 21 rotation values to find the amplitude and phase of the interaction. This yields the simulated map of the NMR signal amplitude and phase for every pixel in Fig. 4b and c. Because the proton positions are randomly generated, the simulated interaction amplitudes are not homogeneous across the chip and are subject to fluctuations. This leads to noise in the final outcome of the simulation.

Supplementary Note 9

NMR Sensitivity

To estimate the NMR sensitivity, we record an NMR measurement with 100 averages. After loading the data, we detrend the time domain signal by subtracting the mean before applying an optimal exponential filter ($e^{-t/\tau}$). The optimal filter parameter τ is obtained by sweeping different values for τ and maximizing the resulting SNR (Supplementary Figure 14, left). After Fourier transformation, we compute the NMR SNR for a $\sim$10 µm×10 µm (30×30 px) region below the microfluidic channel (Supplementary Figure 14, right). This results in a ($\sim$10 µm)^3 NV sensor volume for our $\sim$10 µm NV layer thickness. From our observed SNR of 10.8 (100 averages), we conclude that we would reach an SNR of 3 after 100/13.0 $\approx$ 8 averages. We want to note that optimizing the sensitivity has not been the goal of the current study. As a consequence, we expect large improvements due to future developments (see Supplementary Note 10).

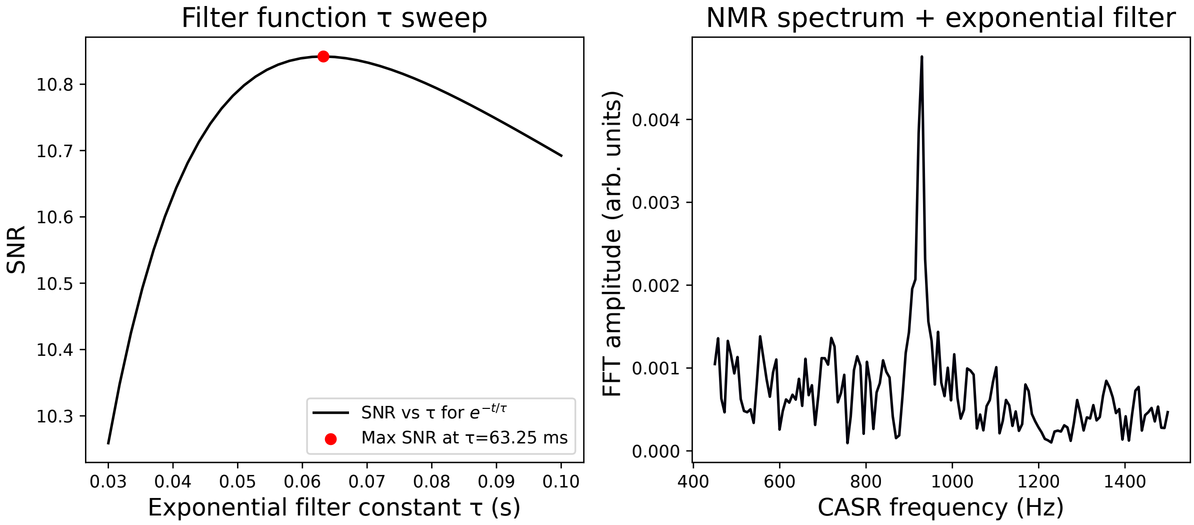

Supplementary Figure 14: NMR sensitivity. Left: SNR of the NMR measurement for different exponential filter parameters τ. Right: NMR amplitude spectrum with the optimized filter applied.

Supplementary Note 10

Projected sensitivity and projected spatial resolution

The primary objective of the current study was to demonstrate the proof of concept of the OMRM technique. Therefore, we did not focus on optimizing sensitivity, which has a large potential for improvement. In Supplementary Table 4 we discuss feasible ways to increase the sensitivity.

Supplementary Table 4: Avenues for sensitivity improvement.

Parameter	Method	Description and evaluation	Improvement factor over current work	Feasibility
**NV readout fidelity**	Improved fluorescence light collection	Fluorescence collection efficiency using a solid immersion lens made of diamond[14].	$\sqrt{8}$-fold	+
	Rabi contrast	Current Rabi contrast is low (∼5%). Reaching >10% for ensembles is feasible[15].	2-fold	+
	Quantum logic enhanced readout	Successfully demonstrated with NV-ensembles[16]. Implementation on a camera has not yet been shown.	6-fold	0
**Diamond material**	Optimized NV density and NV properties	Co-doping with non-nitrogen n-type dopants has shown to improve NV center conversion efficiencies, charge state stability and coherence times[17,18].	No short term improvement anticipated.	-
**Camera**	Quantum efficiency	Increasing the quantum efficiency to >90%[19].	$\sqrt{3}$-fold	0
	Frame rate	Increasing the framerate to 20 kHz (Supplementary Note 4).	$\sqrt{3}$-fold	+
**Magnetic field strength and uniformity**	Increased magnetic field	1 Tesla is feasible using MW resonators and optimized pulse sequences. This is primarily an engineering endeavor[6,8,20].	10-fold	0
	Improving magnetic field uniformity	Removing magnetic parts from the experiments.*	10-fold	+

*Our current ^{1}H T_2^* of ∼60 ms is much lower than the repetition rate of our experiment (3 s in our experiment). Without the objective we have measured a T_2^* of ∼300 ms with this magnet. Thus, a weak residual magnetization of the objective is likely causing magnetic field inhomogeneities, reducing the linewidth and consequently the SNR. Custom design of an objective might be necessary to mitigate this issue.

Increasing the spatial resolution of OMRM by reducing the NV layer thickness will result in a corresponding decrease in the NMR signal's SNR. As extensively discussed by Bruckmaier et al.[10], the strength of the NMR signal remains constant when the sensor-to-sample geometry is kept fixed. However, due to the scaling of the number of interrogated NV centers with the sensor volume V, that is, the overlap of the excitation laser and the NV layer thickness, the sensor's sensitivity scales with $\sqrt{V}$. Supplementary Figure 15 displays (1) the present SNR per square root time (2) the projected sensitivity without Overhauser Dynamic Nuclear Polarization (ODNP) and (3) the projected SNR per square root time (without ODNP) resulting from the technical improvements outlined in Supplementary Table 4. The diagonal lines illustrate the potential changes in SNR per square root time as a function of sensor volume, thereby affecting the spatial resolution of OMRM. We note that at very high spatial resolutions (∼few micrometers), statistical spin polarization can be dominant, which may obscure the thermal NMR signal[6].

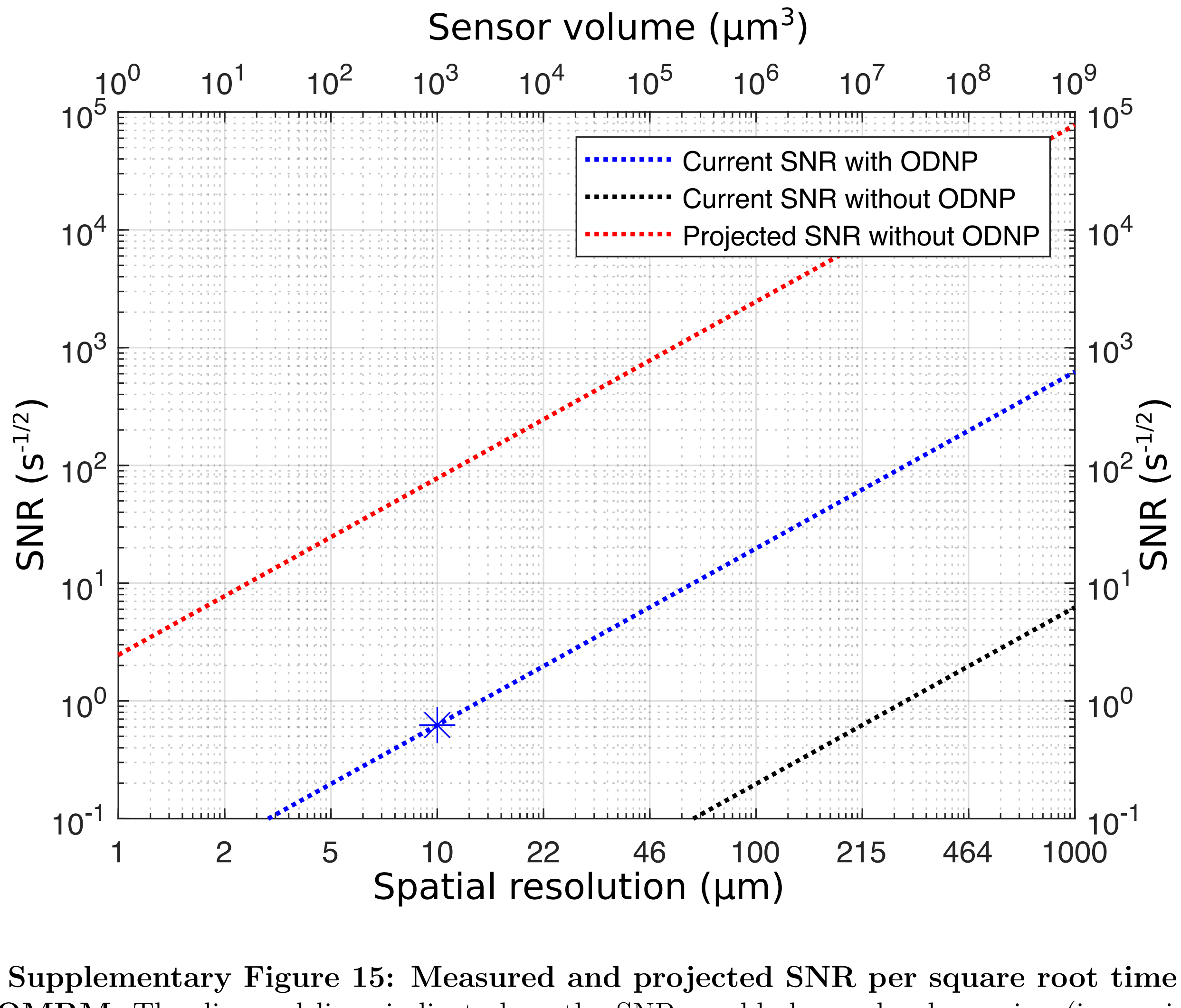

Supplementary Figure 15: Measured and projected SNR per square root time of OMRM. The diagonal lines indicate how the SNR would change by decreasing (increasing) the sensor volume, and thereby increasing (decreasing) the achieved spatial resolution. The blue star corresponds to the current NMR signal SNR per square root time for a (10x10x10) µm^3 sensor volume (Supplementary Note 9).

Supplementary Note 11

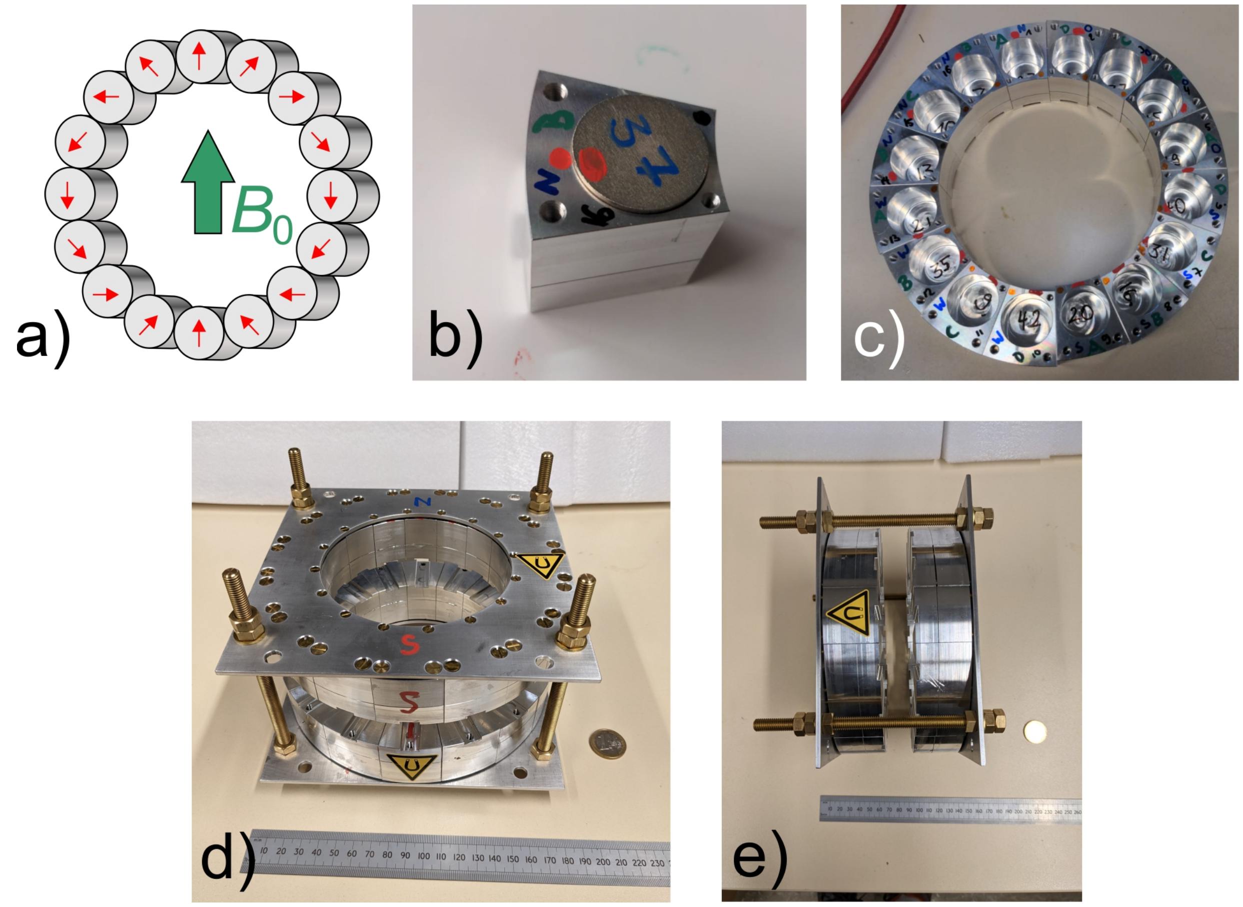

Supplementary Figure 16: Custom-built permanent magnet. **a**, Principle arrangement of 16 cylindrical permanent magnet cylinders (gray) into a single Halbach ring to produce a homogeneous magnetic field in the transverse direction.The red arrows show the individual magnetization of each permanent magnet. **b**, To achieve this individual magnetization direction, each magnet is glued into an aluminum support, while being oriented by an external magnetic field (not shown). **c**, Sixteen such segments form a single ring, two of which are then combined to form the final magnet (**d**, top view, **e**, side view).

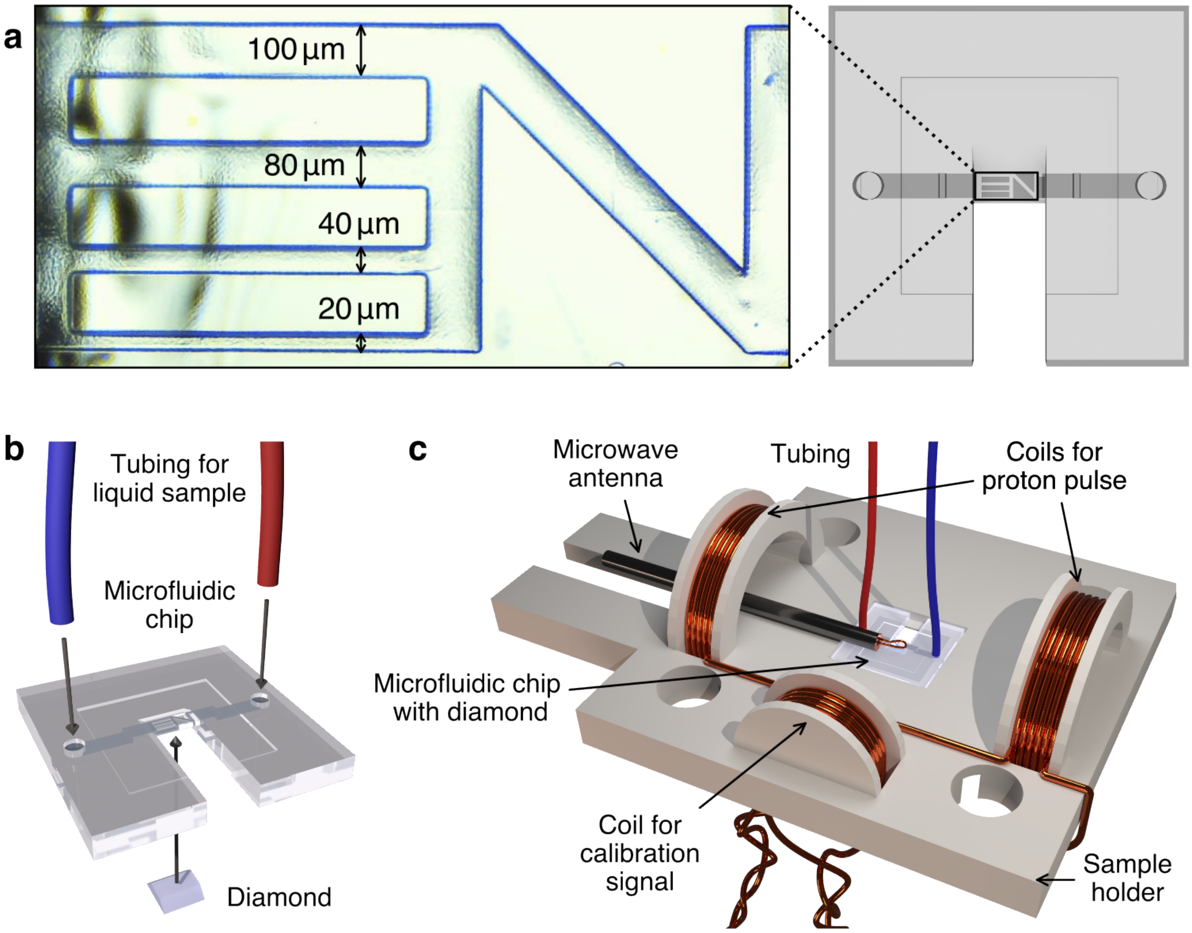

Supplementary Figure 17: Microfluidic chip design and sample holder. **a**, The chip's central structure (on top of the diamond) contains a diagonal channel (∼100 μm width) and four parallel channels with ∼100, 80, 40 and 20 µm width and ∼80 µm height. **b**, The diamond is glued into the microfluidic chip such that it forms the bottom and seals the microfluidic channels. Two liquid sample delivery tubes are glued into the openings provided. **c**, Schematic fully assembled sample holder with microfluidic chip.

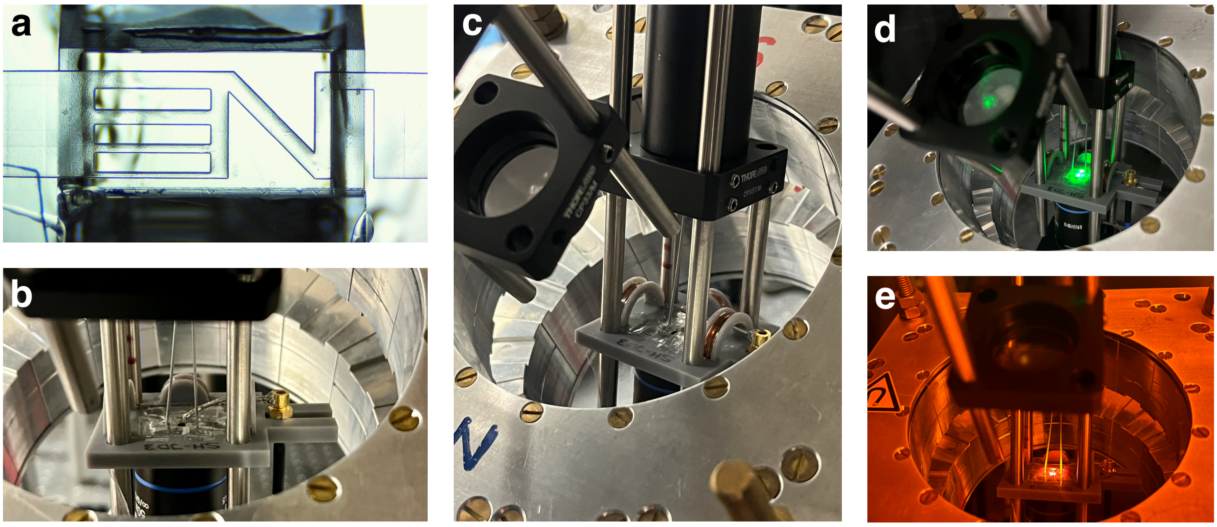

Supplementary Figure 18: Photographs of the probe head. **a**, Optical microscope image of the diamond-microfluidic assembly. **b-e**, Various viewing angles of the assembled sample holder within the fully operational setup.

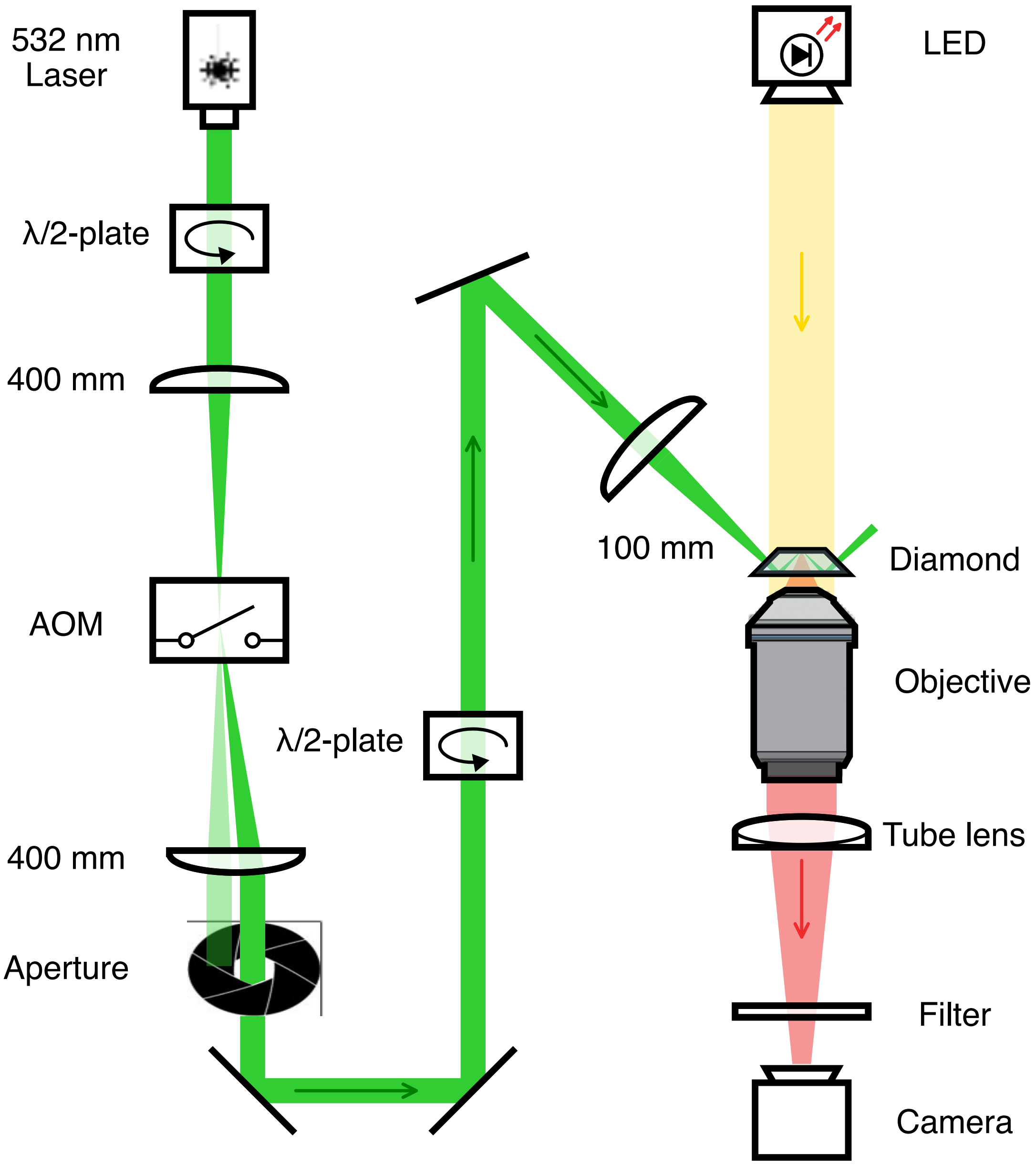

Supplementary Figure 19: OMRM - optics. Figure components adapted from Draeger[21].

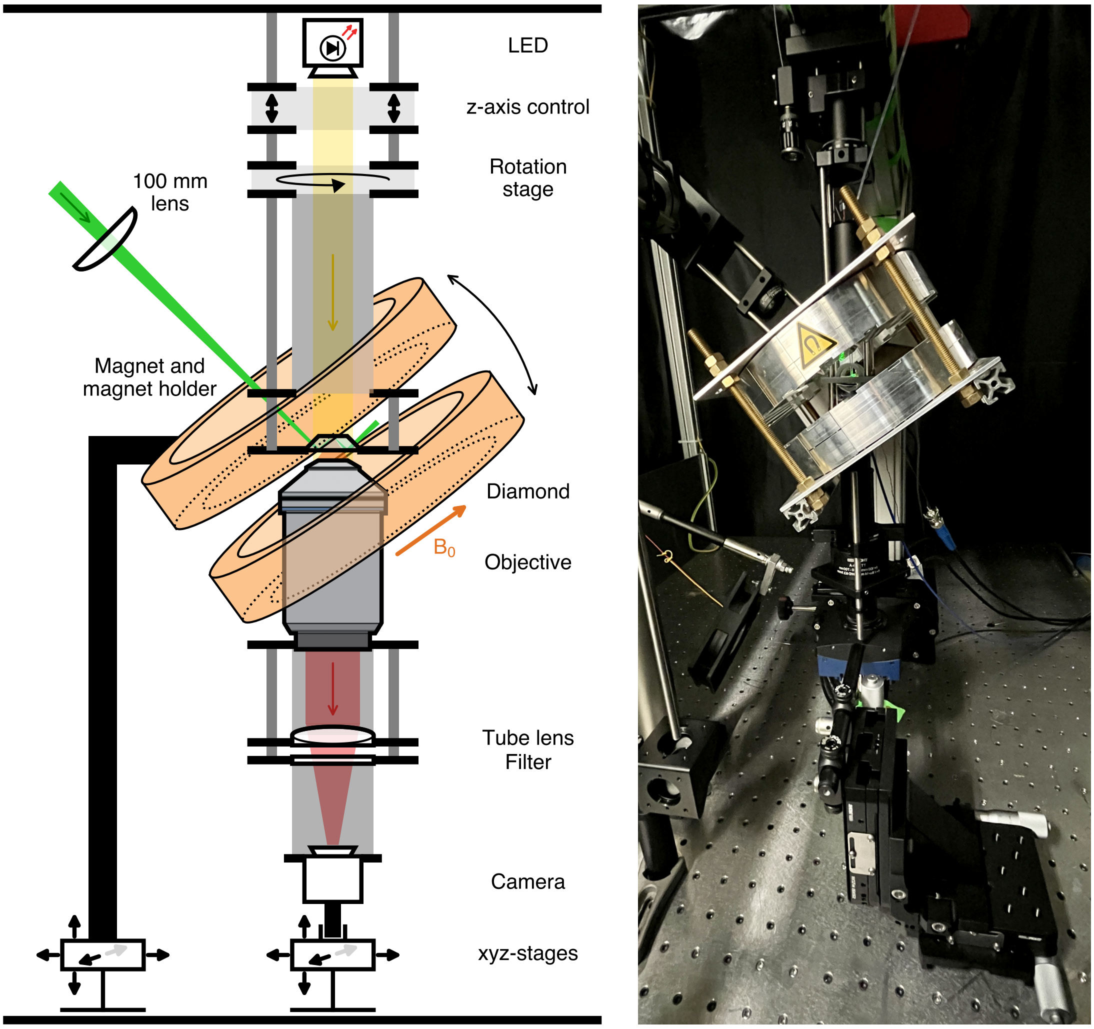

Supplementary Figure 20: OMRM experimental setup. The OMRM microscope consists of three separate parts. The first part is a 30 mm cage system mounted hanging from a frame made of 66 mm optical rails. The assembled sample holder (including the microfluidic chip and NV-diamond) is attached to the end of the cage system, which allows rotation and vertical positioning using a rotation and a z-stage. The LED is attached to the top of the cage system. Second, the magnet (orange) is attached to a rotation stage, which is mounted on an xy-stage on top of a heavy-duty jack. This allows the magnet to be oriented (typically to $\sim 36°$) or moved along all three spatial dimensions, which is critical for optimizing the magnet's position for maximum magnetic field homogeneity. The third part is the imaging optics, consisting of a 50x objective, a long-pass filter, a tube lens, and the camera. The imaging assembly is mounted on a xyz-stage for positioning relative to the diamond sensor. Figure components adapted from Draeger[21].

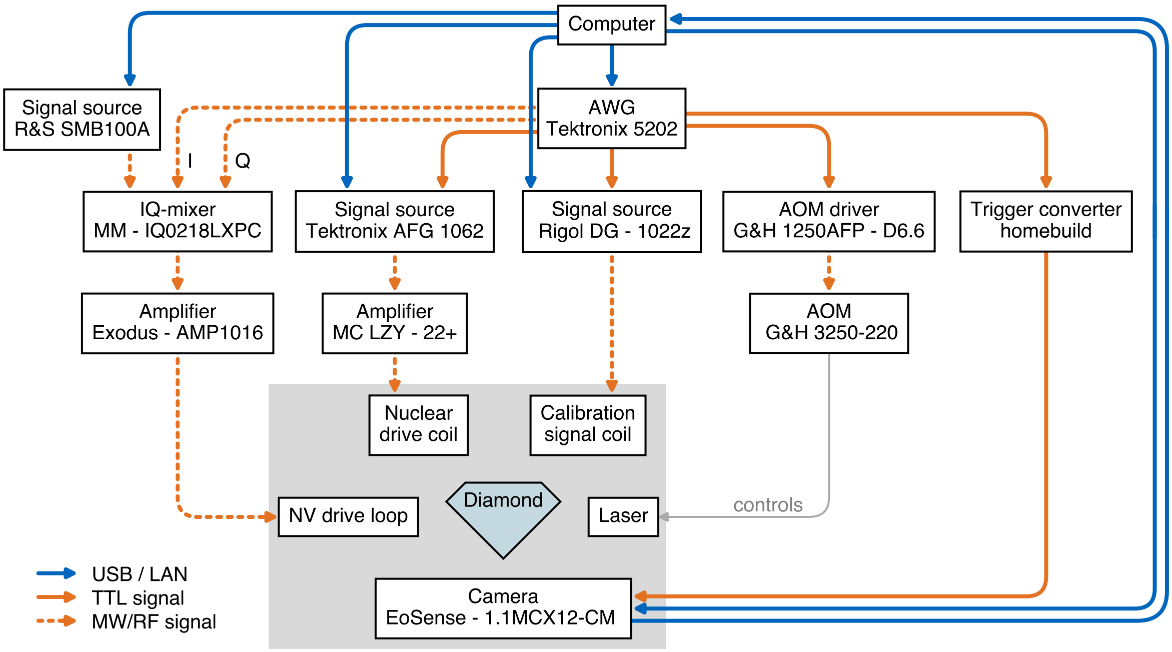

Supplementary Figure 21: OMRM - electronics. Figure components adapted from Draeger[21].

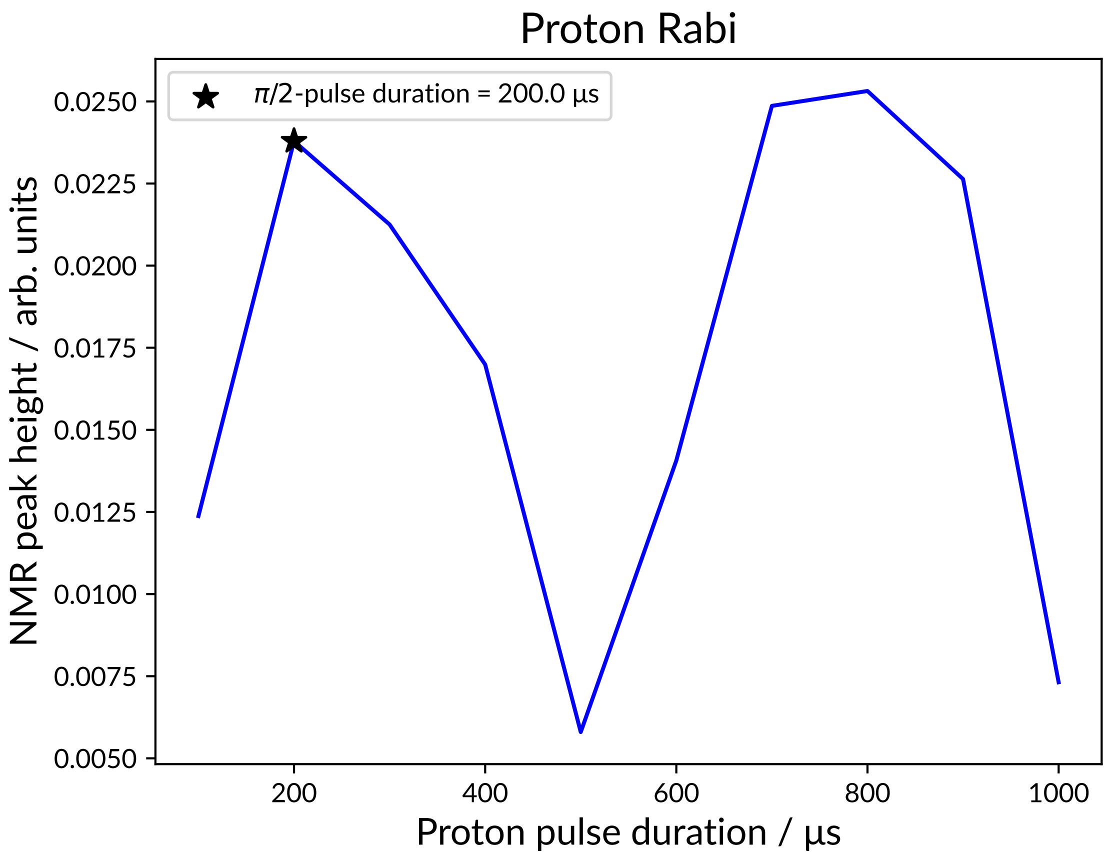

Supplementary Figure 22: Proton Rabi. The duration of the radio frequency pulse, used to excite the NMR signal, is swept in duration and the resulting amplitude of the NMR signal tracked. The $\frac{\pi}{2}$-pulse duration is extracted as the first peak in the signal amplitude.

Supplementary Note 12

Quantum Sensing Measurement Parameters

NV-ESR pulse sequence.

The ESR experiment is used to find the NV center's resonance frequency for the spin state transition $|0\rangle \rightarrow |+1\rangle$. After the initializing laser pulse (532 nm, ∼3 W (after AOM), ∼10 µs to 25 µs), a MW pulse (∼20 µs, swept from 5.20 GHz to 5.22 GHz) is applied before a second laser pulse (∼ 3 µs to 15 µs) is used to read out the NV-fluorescence at ∼6,000 fps over a field of view of ∼ 500 × 700 pixels. The measurements are referenced to a second measurement without a MW pulse for common mode noise rejection. This results in a dip in fluorescence intensity at the resonance condition (∼5.2 GHz, corresponding to $|0\rangle \rightarrow |+1\rangle$ at ∼84 mT).

Magnetic field alignment.

The NV center has 4 possible orientations of its principal axis inside the diamond lattice. For optimal sensitivity, we align the B_0 magnetic field along one of the NV center's principal axes. The orientation of the diamond sensor relative to the magnetic field is adjusted (Supplementary

Figure 20) until ESR resonance lines of the remaining 3 orientations overlay (~3.8 GHz at ~84 mT).

NV-Rabi pulse sequence.

The Rabi experiment is used to determine the control parameters ($\frac{\pi}{2}$ and π-pulses) for the quantum sensing pulse sequences. The experiment consists of an initializing laser pulse (532 nm, ~3 W (after AOM), typ. ≤20 µs), followed by a MW pulse at the NV resonance frequency (~5.2 GHz; $|0\rangle \rightarrow |+1\rangle$ at ~84 mT) with increasing MW pulse durations (~1 ns to 80 ns) before a second laser pulse (~3 µs to 15 µs) is applied to read out the spin state at 6,000 fps over a field of view of $\sim 500 \times 700$ pixels. The measurements are referenced to a second measurement without a MW pulse for common mode noise rejection. The resulting Rabi oscillations are fitted to determine the $\frac{\pi}{2}$ and π-pulse durations (π-pulse durations are typically in the range of ~30 ns to 80 ns). The data in Fig. 2 consists of 1,500 averages. Measuring Rabi images allows optimization of the MW field homogeneity over the field of view by adjusting the MW antenna position. This facilitates homogeneous control of the NV centers over the imaged area.

NV-CASR pulse sequence.

The coherently averaged synchronized readout (CASR) experiment is used for high spectral resolution RF measurements[8]. After bringing the NV center's spin state into a superposition of $|0\rangle$ and $|+1\rangle$ by applying a $\frac{\pi}{2}$-pulse, it becomes maximally sensitive to changes in the magnetic field. The NV center's spin accumulates a phase, which can be translated into a change in fluorescence intensity by applying another $\frac{\pi}{2}$-pulse. While a static offset in the magnetic field leads to an increasing phase accumulation, the oscillating nature of an RF or NMR signal will lead to an inefficient phase accumulation. To counteract this effect, we apply a precisely timed train of π pulses (Dynamical Decoupling sequence - DD) at twice the rate of the RF/NMR signal frequency (typ. π-pulse duration: 30 ns to 80 ns). For our NMR signal at ~3.56 MHz, the spacing between the π-pulses is ~140 ns (denoted as $2{\cdot}\tau$ where $\tau \sim 70$ ns in our experiment). The π-pulse train / DD sequence acts as a rectifying function of the RF signal when applied at its inflection points, causing the NV center spin to accumulate more phase with each RF oscillation. When the π-pulses are applied at the maxima and minima of the RF oscillation, they suppress phase accumulation. The effectiveness of such DD sequences in enabling phase accumulation is thus highly dependent on the phase of the RF signal relative to the DD sequence. A final $\frac{\pi}{2}$-pulse is applied to map the phase accumulation on the NV's spin state population, which can be read out by the fluorescence intensity. Precisely timed repetition of this measurement sequence leads to a periodically varying efficacy in phase accumulation and, thus, fluorescence intensity, determined by the difference in repetition rate and RF signal frequency. The fluorescence oscillates over the readouts at an aliased frequency of the RF signal (Supplementary Figure 3). We term the observed frequency "CASR frequency". For common mode noise rejection, we record a reference measurement by changing the phase of the last $\frac{\pi}{2}$-pulse by 180°. The measurements are referenced according to (signal-reference)/(signal+reference). By tuning the relative phase of the π pulses, the DD sequence can be made robust against pulse errors. One popular robust DD sequence is the so-called XY8 sequence, consisting of 8 phase-shifted π-pulses. In our experiments, we typically repeat the XY8 blocks 10-20 times per measurement to balance phase accumulation and the decoherence of our quantum state.

Sensitivity estimation and RF calibration.

To calibrate the sensitivity of our experiment, a calibration signal of known size is supplied to the system using a wound copper wire coil next to the sample. Sweeping the power of the calibration signal allows tracking of the accumulated phase of the NV center's spin. We reconstruct the magnetic field amplitude of the calibration RF signal from the measured dependence of the phase accumulation of the RF input power[8]. In our experiments, an RF amplitude of ∼20 mVpp corresponds to a ∼10 nT signal.

NV-CASR pulse sequence for ^{1}H NMR samples.

To detect the ^{1}H NMR signal, a proton $\frac{\pi}{2}$-pulse (duration: ∼200 µs, power: ∼70% of NV-drive amplitude, frequency: ∼3.5625 MHz) is applied to start the precession of the nuclear spins. After initial detection of the NMR signal, the proton pulse time is swept to calibrate its optimal duration (Supplementary Figure 22). To increase the signal amplitude and reduce the averaging time, the sample is hyperpolarized using an Overhauser DNP pulse (duration: ∼400 ms, frequency: ∼2.34 GHz), which transfers the thermal polarization of electron spins in TEMPOL to the nuclear spins[22]. As the effectiveness of the Overhauser DNP drive improves with increased power and duration, these parameters are heuristically selected as a balanced compromise to maximize efficiency while avoiding overheating and potential damage to the microwave equipment. The magnet's position was optimized for the most homogeneous magnetic field area. To verify that the NMR signal is assigned correctly, the NMR measurements were repeated with a frequency offset in the Overhauser DNP pulse and without a proton $\frac{\pi}{2}$-pulse (in both cases, the NMR signal disappears). The ^{1}H resonance frequency is very sensitive to changes in the magnetic field strength. To minimize the effects of slow magnetic field drifts, most likely caused by changes in temperature, long measurements were saved in chunks of 100 averages (∼5 min) to prevent excessive line broadening of the NMR signal (see). Overall, the data presented in Fig. 3 and 4 were averaged ∼ 17,000 to 19,000 times.

Supplementary Note 13

Software

Measurement software.

We wrote custom lab software (QuPyt) to facilitate:

- Configuration and on the fly parameter updates of measurement devices.
- Experiment timing / synchronization.
- Live data processing.
- Reusable pulse sequence design and upload.
- General purpose measurement configuration.
- Measurement automatization.

The code is available open source on GitHub at https://github.com/KarDB/QuPyt. A paper describing the software is in preparation.

Data analysis software.

To analyze large amounts of data (multiple mega- to gigabytes per measurement) by fitting up to 1 million cosines or Lorentzian curves per measurement, we wrote portions of our analysis code in Rust. The Rust modules are compiled into Python wheels and can be installed and used from within a Python environment. The code is open source and available on GitHub (QuFit-rs) at https://github.com/KarDB/QuFit-rs.

References

1. DeVience, S. J. *et al.* Nanoscale NMR spectroscopy and imaging of multiple nuclear species. *Nature Nanotechnology* **10,** 129–134 (2 Feb. 1, 2015).
2. Ziem, F., Garsi, M., Fedder, H. & Wrachtrup, J. Quantitative nanoscale MRI with a wide field of view. *Scientific Reports* **9** (2019).
3. Liu, K. S. *et al.* Surface NMR using quantum sensors in diamond. *Proc Natl Acad Sci U S A* **119** (Feb. 2022).
4. Degen, C. L., Reinhard, F. & Cappellaro, P. Quantum sensing. *Rev. Mod. Phys.* **89,** 035002. `https://link.aps.org/doi/10.1103/RevModPhys.89.035002` (3 July 2017).
5. Levine, E. V. *et al.* Principles and techniques of the quantum diamond microscope. *Nanophotonics* **8,** 1945–1973. `https://www.degruyter.com/view/journals/nanoph/8/11/article-p1945.xml` (Nov. 1, 2019).
6. Allert, R. D., Briegel, K. D. & Bucher, D. B. Advances in nano- and microscale NMR spectroscopy using diamond quantum sensors. *Chem. Commun.* **58,** 8165–8181. `http://dx.doi.org/10.1039/D2CC01546C` (59 2022).
7. Bucher, D. B. *et al.* Quantum diamond spectrometer for nanoscale NMR and ESR spectroscopy. *Nature Protocols* **14** (Sept. 1, 2019).
8. Glenn, D. R. *et al.* High-resolution magnetic resonance spectroscopy using a solid-state spin sensor. *Nature* **555** (7696 Jan. 3, 2018).
9. Nishimura, S., Tsukamoto, M., Sasaki, K. & Kobayashi, K. *Investigations of optical aberration on quantum diamond microscopy toward high spatial resolution and sensitivity* 2024. arXiv: `2402.14422 [physics.optics]`. `https://arxiv.org/abs/2402.14422`.
10. Bruckmaier, F., Briegel, K. D. & Bucher, D. B. Geometry dependence of micron-scale NMR signals on NV-diamond chips. *Journal of Magnetic Resonance Open* **8-9,** 100023. ISSN: 2666-4410. `https://www.sciencedirect.com/science/article/pii/S2666441021000145` (2021).
11. Sokolenko, S. *et al.* Robust 1D NMR lineshape fitting using real and imaginary data in the frequency domain. *Journal of Magnetic Resonance* **298,** 91–100. ISSN: 1090-7807. `https://www.sciencedirect.com/science/article/pii/S1090780718303057` (2019).
12. Keeler, J. *Understanding NMR spectroscopy* (John Wiley & Sons, 2010).
13. Haase, S.-H., Dmitriev, A. & Staikopoulos, M. *bvh: A fast BVH using SAH* 2024. `https://github.com/svenstaro/bvh`.
14. Siyushev, P. *et al.* Monolithic diamond optics for single photon detection. *Applied Physics Letters* **97.** ISSN: 1077-3118. `http://dx.doi.org/10.1063/1.3519849` (Dec. 2010).

15. Edmonds, A. M. *et al.* Characterisation of CVD diamond with high concentrations of nitrogen for magnetic-field sensing applications. *Materials for Quantum Technology* **1,** 025001. ISSN: 2633-4356. `http://dx.doi.org/10.1088/2633-4356/abd88a` (Mar. 2021).

16. Arunkumar, N. *et al.* Quantum Logic Enhanced Sensing in Solid-State Spin Ensembles. *Physical Review Letters* **131.** ISSN: 1079-7114. `http://dx.doi.org/10.1103/PhysRevLett.131.100801` (Oct. 2023).

17. Radishev, D. *et al.* Investigation of NV centers charge states in CVD diamond layers doped by nitrogen and phosphorous. *Journal of Luminescence* **239,** 118404. ISSN: 0022-2313. `http://dx.doi.org/10.1016/j.jlumin.2021.118404` (Nov. 2021).

18. Watanabe, A. *et al.* Shallow NV centers augmented by exploiting n-type diamond. *Carbon* **178,** 294–300. ISSN: 0008-6223. `http://dx.doi.org/10.1016/j.carbon.2021.03.010` (June 2021).

19. Wang, Y. *et al.* Quantitative performance evaluation of a back-illuminated sCMOS camera with 95% QE for super-resolution localization microscopy. *Cytometry Part A* **91,** 1175–1183. ISSN: 1552-4930. `http://dx.doi.org/10.1002/cyto.a.23282` (Nov. 2017).

20. Neuling, N. R., Allert, R. D. & Bucher, D. B. Prospects of single-cell nuclear magnetic resonance spectroscopy with quantum sensors. *Current Opinion in Biotechnology* **83,** 102975. ISSN: 0958-1669. `http://dx.doi.org/10.1016/j.copbio.2023.102975` (Oct. 2023).

21. Draeger, J. C. *NMR Microscopy using Quantum Sensors in Diamond* MA thesis (Technical University of Munich, Munich, Germany, 2022).

22. Bucher, D. B., Glenn, D. R., Park, H., Lukin, M. D. & Walsworth, R. L. Hyperpolarization-Enhanced NMR Spectroscopy with Femtomole Sensitivity Using Quantum Defects in Diamond. *Phys. Rev. X* **10,** 021053. `https://link.aps.org/doi/10.1103/PhysRevX.10.021053` (2 June 2020).
